# Coupling of α,α-difluoro-substituted organozinc reagents with 1-bromoalkynes

**DOI:** 10.3762/bjoc.11.231

**Published:** 2015-11-10

**Authors:** Artem A Zemtsov, Alexander D Volodin, Vitalij V Levin, Marina I Struchkova, Alexander D Dilman

**Affiliations:** 1 N. D. Zelinsky Institute of Organic Chemistry, 119991 Moscow, Leninsky prosp. 47, Russian Federation; 2 Higher Chemical College, Russian Academy of Sciences, 125047 Moscow, Miusskaya sq. 9, Russian Federation

**Keywords:** 1-bromoalkynes, cross-coupling, organofluorine compounds, organozinc reagents

## Abstract

α,α-Difluoro-substituted organozinc reagents generated from conventional organozinc compounds and difluorocarbene couple with 1-bromoalkynes affording *gem*-difluorinated alkynes. The cross-coupling proceeds in the presence of catalytic amounts of copper iodide in dimethylformamide under ligand-free conditions.

## Introduction

*gem*-Difluorinated organic compounds have attracted increasing attention nowadays due to their applicability in medicinal chemistry [1–2] and other fields. Indeed, unique stereoelectronic properties of the CF_2_-unit may be exploited in conformational analysis [3–5], carbohydrate and peptide research [6–7], and reaction engineering [8–9].

Typically, the difluoromethylene fragment is created by deoxyfluorination, which requires harsh or hazardous conditions [10–11]. Alternatively, functional group manipulations starting from available CF_2_-containing building blocks can be considered, but multistep sequences render this approach laborious [12–14]. Difluoro-substituted cyclopropanes and cyclopropenes constitute a specific class of compounds accessible by difluorocarbene addition to multiple bonds [15].

**Scheme 1:**
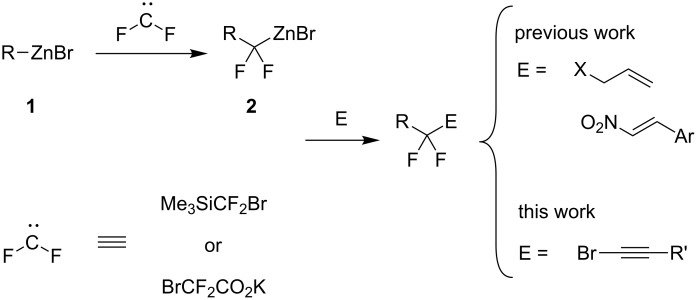
Reaction of organozinc compounds.

Recently, we proposed a general method for assembling *gem*-difluorinated structures from organozinc reagents **1**, difluorocarbene, and a terminating electrophile [16–21] (Scheme 1). (Bromodifluoromethyl)trimethylsilane [16–18] or potassium bromodifluoroacetate [19] can be used as precursors of difluorocarbene. In this process, the use of C-electrophiles is particularly important since it allows for the formation of two C–C bonds within one experimental run. Previously, as C-electrophiles in this methodology, only allylic substrates [17] and nitrostryrenes (with the NO_2_ serving as a leaving group) [20], were employed. Herein, we report that 1-bromoalkynes, which are known to be involved in reactions with various organometallic compounds [22–27], can be used as suitable coupling partners for difluorinated organozinc compounds **2**. This reaction provides straightforward access to α,α-difluorinated alkynes [13–14,28–31]. Our method is based on facile zinc/copper exchange allowing for versatile couplings described for non-fluorinated organozinc compounds [32–37].

## Results and Discussion

Organozinc compound **2a** generated from benzylzinc bromide was first evaluated in a reaction with haloalkynes derived from phenylacetylene (Table 1). First, most reactive iodo-substituted alkyne **3a-I** (X = I) was evaluated in the presence of copper iodide (10 mol %). Expected product **4a** was formed in 12% yield, but its yield was tripled simply by adding 2 equiv of DMF additive (Table 1, entries 1 and 2). However, in these experiments, the reaction mixtures contained about 40% of (2,2-difluoro-2-iodoethyl)benzene (PhCH_2_CF_2_I) arising from zinc/iodine exchange between **2a** and the iodoalkyne. Chloroalkyne **3a-Cl** was markedly less reactive, likely because of the strong carbon–chlorine bond. Fortunately, bromoalkyne **3a**-**Br** provided the best results, with the optimal conditions involving the use of DMF as a solvent and only 5 mol % of copper iodide at 0 °C to room temperature, which afforded the coupling product in 79% isolated yield (Table 1, entry 5). The addition of various ligands, as well as the use of other copper salts, did not had a beneficial effect.

**Table 1:** Optimization studies.

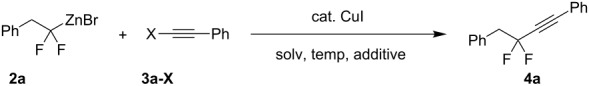							

Entry	X	**2a** (equiv)	Conditions	Solvent	CuI (equiv)	Additive (equiv)	Yield of **4a**, %^a^

1	I	2	−50 °C → rt; 4 h at rt	MeCN	0.1	–	12
2	I	1.3	−50 °C → rt; 4 h at rt	MeCN	0.1	DMF (2)	35
3	Cl	2	0 °C → rt; 16 h at rt	MeCN	0.1	DMF (2)	32
4	Br	1.5	0 °C → rt; 16 h at rt	MeCN	0.1	DMF (2)	60
5	Br	1.5	0 °C → rt; 16 h at rt	DMF	0.05	–	79^b^

^a^Determined by ^19^F NMR with internal standard. ^b^Isolated yield.

Under the optimized conditions, a series of organozinc compounds **2** were coupled with bromoalkynes **3** (Table 2). Good yields of coupling products **4** were typically achieved. The reaction tolerates ester groups or TBS-protected hydroxy groups. Aromatic iodide also remains unaffected (Table 2, entry 2).

**Table 2:** Reaction of organozinc compounds **2** with bromoalkynes **3**.

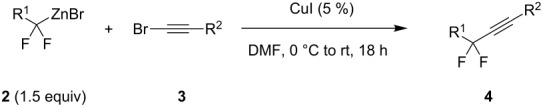				

Entry	**2**	**3**	**4**	Yield of **4**, %^a^

1	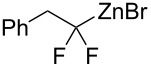 **2a**	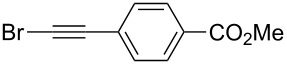 **3b**	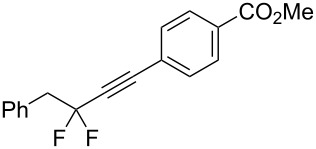 **4b**	84
2	**2a**	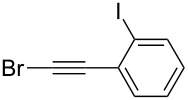 **3c**	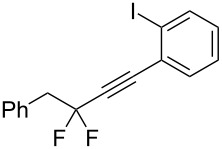 **4c**	82
3	**2a**	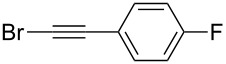 **3d**	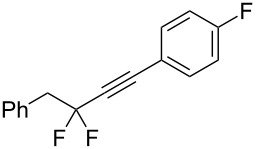 **4d**	70
4	**2a**	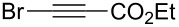 **3e**	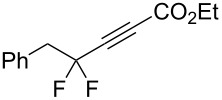 **4e**	84
5	**2a**	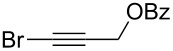 **3f**	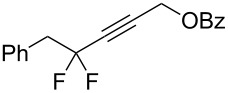 **4f**	67
6^b^	**2a**	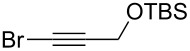 **3g**	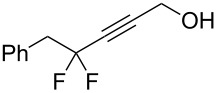 **4g**	80
7^b^	**2a**	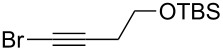 **3h**	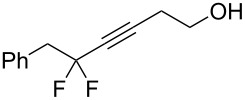 **4h**	75
8	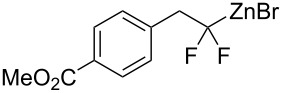 **2b**	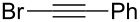 **3a-Br**	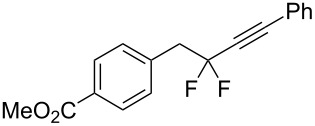 **4i**	80
9	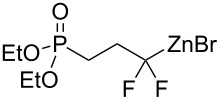 **2e**	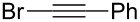 **3a-Br**	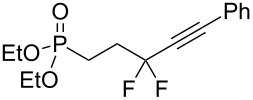 **4j**	81
10	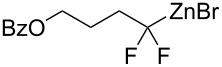 **2c**	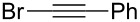 **3a-Br**	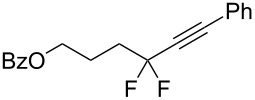 **4k**	72
11^b^	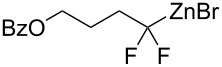 **2c**	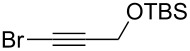 **3g**	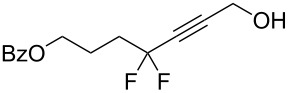 **4l**	71
12^b^	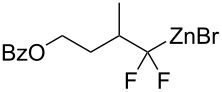 **2d**	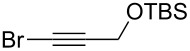 **3g**	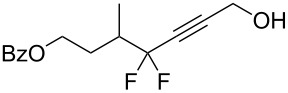 **4m**	62

^a^Isolated yield. ^b^The crude product was desilylated.

**Scheme 2:**
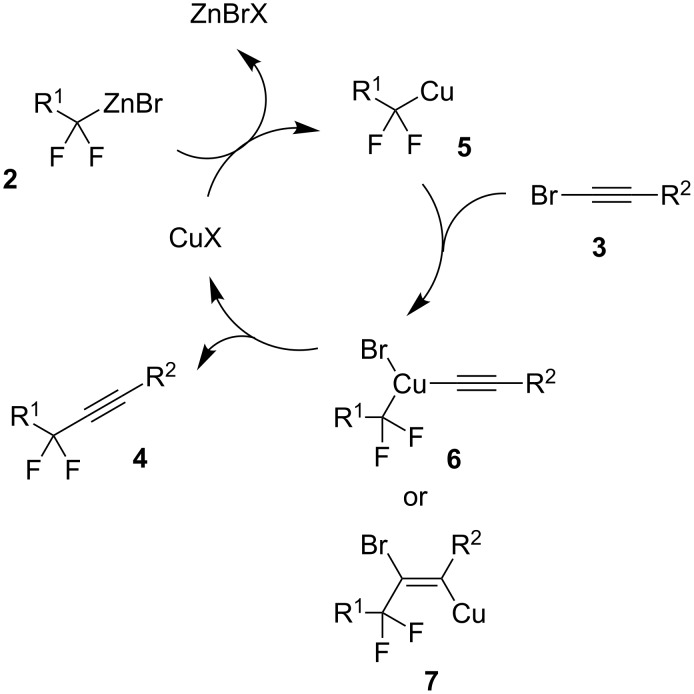
Proposed mechanism.

As for the mechanism, we believe that the reaction starts with the zinc/copper exchange resulting in the formation of fluorinated organocopper species **5** (Scheme 2). Compound **5** interacts with bromoalkyne **3** either by oxidative addition generating copper(III) intermediate **6** or by triple bond carbometallation [38] generating copper(I) intermediate **7**. Subsequent reductive elimination (from **6**) or β-elimination (from **7**) leads to the product and regenerates the copper(I) catalyst.

## Conclusion

In summary, a method for the copper-catalyzed coupling of α,α-difluoro-substituted organozinc compounds with 1-bromoalkynes has been developed. The reaction is performed under mild conditions affording *gem*-difluoro-substituted alkynes in good yields.

## Supporting Information

File 1 Full experimental details, compound characterization, and copies of NMR spectra.
