# Supplementary material for: Coupling of α,α-difluoro-substituted organozinc reagents with 1-bromoalkynes
Source: Beilstein J Org Chem. 2015 Nov 10;11:2145–9. doi: 10.3762/bjoc.11.231 (PMC4660974; doi:10.3762/bjoc.11.231)
Supplement: File 1 — Full experimental details, compound characterization, and copies of NMR spectra. [file Beilstein_J_Org_Chem-11-2145-s001.pdf]

Supporting information
for
Coupling of α,α-difluoro-substituted organozinc reagents with 1-bromoalkynes

Artem A. Zemtsov[1], Alexander D. Volodin[1,2], Vitalij V. Levin[1], Marina I. Struchkova[1]

and Alexander D. Dilman[1,*]

Address: [1]N. D. Zelinsky Institute of Organic Chemistry, 119991 Moscow, Leninsky prosp. 47, Russian Federation and [2]Higher Chemical College, Russian Academy of Sciences, 125047 Moscow, Miusskaya sq. 9, Russian Federation

Email: Alexander D. Dilman - adil25@mail.ru

*Corresponding

Full experimental details, compound characterization, and copies of NMR spectra

General

All reactions were performed under an argon atmosphere. THF was distilled under argon from $LiAlH_4$ prior to use. DMF was distilled under argon from P_2O_5 and stored over MS 4 Å. CH_3CN was distilled from P_2O_5 and CaH_2 and stored over MS 4 Å. Column chromatography was carried out employing silica gel (230–400 mesh). Precoated silica gel plates F-254 were used for thin-layer analytical chromatography visualizing with UV and/or acidic aq. $KMnO_4$ solution.

NMR spectra were recorded with a Bruker AM-300 instrument. ^{1}H NMR chemical shifts were determined relative to the signal of a residual protonated solvent: $CDCl_3$ (δ = 7.26 ppm). ^{13}C NMR chemical shifts were determined relative to the signal of solvent: $CDCl_3$ (δ = 77.16 ppm). High resolution mass spectra (HRMS) were measured using electrospray ionization (ESI) and a time-of-flight (TOF) mass analyzer. The measurements were done in a positive ion mode (interface capillary voltage–4500 V) or in a negative ion mode (3200 V); mass range from *m*/*z* 50 to *m*/*z* 3000.

Organozinc reagents **1a,b,d** were prepared by the protocol described in our previous work[1]. Reagent **1c** was obtained from 3-bromopropyl benzoate and zinc using the standard procedure[1a] with the reaction time 7 days. Bromoalkynes **3**[2] were prepared by a modified literature protocol.

[1] (a) Levin, V. V.; Zemtsov, A. A.; Struchkova, M. I.; Dilman, A. D. *Org. Lett.* **2013**, *15*, 917–919. (b) Levin, V. V.; Zemtsov, A. A.; Struchkova, M. I.; Dilman, A. D. *J. Fluorine Chem.* **2015**, *171*, 97–101.
[2] Feng, Y.-S.; Xu, Z.-Q.; Mao, L.; Zhang, F.-F.; Xu, H.-J. *Org. Lett.* **2013**, *15*, 1472–1475.

Preparation of bromoalkynes 3b–d.

Triphenylphosphine (30.0 mmol, 7.86 g) was slowly added to a stirred solution of aldehyde (10.0 mmol) and CBr_4 (15.0 mmol, 4.98 g) in dry CH_2Cl_2 (20 mL) at 0 °C. The resulting suspension was stirred at this temperature for 1 hour, warmed to room temperature and then diluted with hexane (40 mL). The mixture was quenched with water (40 mL), then the organic layer was separated and filtered through a shot silica gel pad. The solution was concentrated in vacuum. The resulting 2,2-dibromostyrene was dissolved in CH_2Cl_2 (10 mL) and treated with a solution of KOH (50 mmol, 2.8 g) in water (2 mL). Then, benzyltriethylammonium chloride (2.0 mmol, 0.46 g) was added and the reaction mixture was vigorously stirred for 6 hours at room temperature (the conversion of 2,2-dibromostyrene was monitored by GC analysis). The reaction was quenched with water (40 mL) and extracted with hexane (3 × 20 mL). The combined organic layers were concentrated under vacuum and the residue was purified by column chromatography.

Preparation of bromoalkynes 3a-Br and 3e–h

Silver nitrate (0.1 mmol, 17 mg) was added to a solution of alkyne (10.0 mmol) and *N*-bromosuccinimide (11.0 mmol, 1.96 g) in acetone (10 mL) at room temperature. The reaction was stirred overnight, quenched with water (30 mL) and extracted with hexane (3 × 10 mL). The combined organic layers were concentrated under vacuum and the residue was purified by column chromatography.

General procedure for preparation of reagents 2a,c,e from $BrCF_2CO_2K$

The freshly titrated solution of organozinc reagent **1** (3.0 mmol) in THF was concentrated in vacuum and the resulting viscous residue was dissolved in DMF (4 mL). Then $BrCF_2CO_2K$ (4.5 mmol, 0.96 g) was added and the resulting clear solution was vigorously stirred at 50 ºC for approximately 50 minutes. The conversion was checked by GC analysis of a brominated aliquote. After completion the concentration of **2** was determined by ^{19}F NMR with $PhCF_3$ as an internal standard (~0.45 M).

General procedure for preparation of reagents 2b,d from Me_3SiCF_2Br

A freshly titrated solution of organozinc reagent **1** (3.0 mmol) in THF was concentrated under vacuum and the resulting viscous residue was dissolved in CH_3CN (3 mL). Then, sodium acetate (3.6 mmol, 295 mg) and Me_3SiCF_2Br (3.6 mmol, 731 mg) were added at −20 °C. The reaction mixture was stirred for 18 hours at this temperature and then concentrated under vacuum at 0 °C. The residue was dissolved in DMF (3 mL). The concentration of **2** was determined by ^{19}F NMR with $PhCF_3$ as an internal standard (~0.55 M).

General procedure for coupling reaction

A titrated solution of **2** (1.5 mmol) was cooled to 0 °C with an ice bath and then bromoalkyne **1** (1.0 mmol) and CuI (0.05 mmol, 9.5 mg) were added. The stirred suspension was allowed to slowly warm to room temperature for 2 hours and then stirred for additional 16 hours. The resulting mixture was quenched with aqueous HCl (0.5 M, 6 mL) and extracted with Et_2O (3 × 5 mL). The combined organic layers were concentrated under vacuum.

For acetylenes **3a-Br, 3b-f**, the product **4** was purified by column chromatography.

For acetylenes **3g**,**h**, the residue was dissolved in a mixture of AcOH (3 mL), THF (1 mL) and water (1 mL) and kept for 8 hours at 40 °C. The mixture was diluted with CH_2Cl_2 (10 mL) and carefully washed with water (3 × 10 mL) and saturated Na_2CO_3 solution (10 mL). The organic layer was concentrated under vacuum and the residue was purified by column chromatography.

(3,3-Difluorobut-1-yne-1,4-diyl)dibenzene (4a)

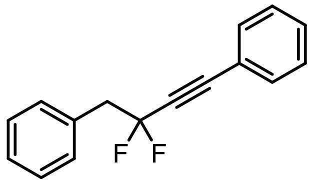

Yield 191 mg (79%). Colorless oil. R_f = 0.33 (Hexane/CH_2Cl_2, 20:1).

^{1}H NMR (300 MHz, $CDCl_3$), δ: 3.51 (t, *J* = 14.2 Hz, 2H), 7.31–7.53 (m, 10H).

^{13}C{^{1}H} NMR (75 MHz, $CDCl_3$), δ: 46.0 (t, *J* = 27.5 Hz), 81.7 (t, *J* = 40.4 Hz), 88.2 (t, *J* = 6.9 Hz), 114.5 (t, *J* = 233.8 Hz), 120.3 (t, *J* = 2.9 Hz), 127.9, 128.5, 128.6, 130.0, 130.8, 132.2 (t, *J* = 2.3 Hz), 132.3 (t, *J* = 4.0 Hz).

^{19}F NMR (282 MHz, $CDCl_3$), δ: –82.4 (t, *J* = 14.2 Hz).

Calcd for $C_{16}H_{12}F_2$ (242.26): C 79.32, H 4.99. Found: C 79.18, H 5.15.

Methyl 4-(3,3-difluoro-4-phenylbut-1-yn-1-yl)benzoate (4b)

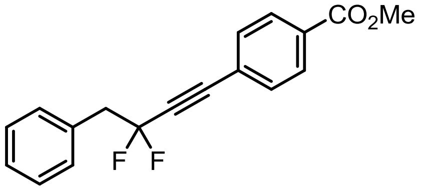

Yield 252 mg (84%). Colorless solid. Mp 64–65 °C. R_f = 0.35 (Hexane/EtOAc, 10:1).

^{1}H NMR (300 MHz, $CDCl_3$), δ: 3.47 (t, *J* = 14.5 Hz, 2H), 3.93 (s, 3H), 7.30–7.42 (m, 5H), 7.48 (d, *J* = 8.3 Hz, 2H), 8.02 (d, *J* = 8.3 Hz, 2H).

^{13}C{^{1}H} NMR (75 MHz, $CDCl_3$), δ: 45.7 (t, *J* = 27.2 Hz), 52.3, 83.9 (t, *J* = 40.7 Hz), 87.0 (t, *J* = 6.9 Hz), 114.2 (t, *J* = 234.5 Hz), 124.6 (t, *J* = 2.6 Hz), 127.9, 128.6, 129.6, 130.7, 131.2, 132.0 (t, *J* = 2.2 Hz), 166.1.

^{19}F NMR (282 MHz, $CDCl_3$), δ: –83.0 (t, *J* = 14.5 Hz).

Calcd for $C_{18}H_{14}F_2O_2$ (300.30): C 71.99, H 4.70. Found: C 71.91, H 4.78.

1-(3,3-Difluoro-4-phenylbut-1-yn-1-yl)-2-iodobenzene (4c)

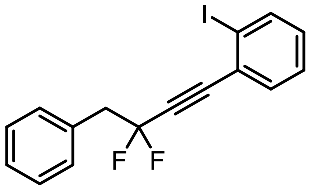

Yield 302 mg (82%). Colorless solid. Mp 73–74 °C. R_f = 0.28 (Hexane/CH_2Cl_2, 10:1).

^{1}H NMR (300 MHz, $CDCl_3$), δ: 3.56 (t, *J* = 14.6 Hz, 2H), 7.09 (td, *J* = 7.8, 1.7 Hz, 1H), 7.30–7.53 (m, 7H), 7.87 (d, *J* = 7.8 Hz, 1H).

^{13}C{^{1}H} NMR (75 MHz, $CDCl_3$), δ: 45.8 (t, *J* = 27.2 Hz), 84.8 (t, *J* = 40.6 Hz), 89.2 (t, *J* = 6.9 Hz), 100.4 (t, *J* = 2.5 Hz), 114.2 (t, *J* = 234.5 Hz), 127.0 (t, *J* = 2.6 Hz), 127.9 (d, *J* = 8.9 Hz), 128.5, 130.9, 131.0, 131.9 (t, *J* = 3.9 Hz), 133.6 (t, *J* = 1.9 Hz), 139.0.

^{19}F NMR (282 MHz, $CDCl_3$), δ: –82.6 (t, *J* = 14.6 Hz).

Calcd for $C_{16}H_{11}F_2I$ (368.16): C 52.20, H 3.01. Found: C 52.05, H 3.07.

1-(3,3-Difluoro-4-phenylbut-1-yn-1-yl)-4-fluorobenzene (4d)

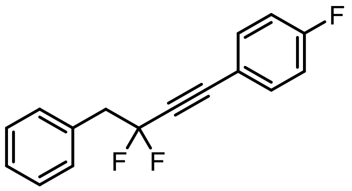

Yield 182 mg (70%). Colorless oil. R_f = 0.31 (Hexane/CH_2Cl_2, 20:1).

^{1}H NMR (300 MHz, $CDCl_3$), δ: 3.49 (t, *J* = 14.2 Hz, 2H), 7.06 (dd, *J* = 8.7 Hz, 2H), 7.35–7.51 (m, 7H).

^{13}C{^{1}H} NMR (75 MHz, $CDCl_3$), δ: 45.9 (t, *J* = 27.5 Hz), 81.5 (td, *J* = 40.7, 1.6), 87.2 (t, *J* = 6.9 Hz), 114.4 (t, *J* = 234.0 Hz), 116.0 (d, *J* = 22.4 Hz), 116.4 (dt, *J* = 3.4, 2.9 Hz), 127.9, 128.5, 130.8, 132.3 (t, *J* = 4.0 Hz), 134.2 (dt, *J* = 8.6, 2.3 Hz), 163.6 (d, *J* = 252.4 Hz).

^{19}F NMR (282 MHz, $CDCl_3$), δ: –108.7 (tt, 1F, *J* = 8.7, 4.8 Hz), –82.5 (t, 2F, *J* = 14.2 Hz).

Calcd for $C_{16}H_{11}F_3$ (260.25): C 73.84, H 4.26. Found: C 73.74, H 4.37.

Ethyl 4,4-difluoro-5-phenylpent-2-ynoate (4e)

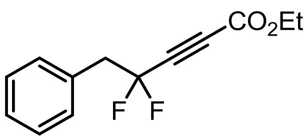

Yield 200 mg (84%). Colorless oil. R_f = 0.33 (Hexane/EtOAc, 15:1).

^{1}H NMR (300 MHz, $CDCl_3$), δ: 1.33 (t, *J* = 7.2 Hz, 3H), 3.39 (t, *J* = 15.1 Hz, 2H), 4.27 (q, *J* = 7.2 Hz, 2H), 7.29–7.40 (m, 5H).

^{13}C{^{1}H} NMR (75 MHz, $CDCl_3$), δ: 14.0, 45.2 (t, *J* = 25.9 Hz), 62.8, 77.8 (t, *J* = 6.8 Hz) 113.1 (t, *J* = 237.0 Hz), 128.2, 128.7, 130.7, 130.8, 151.9 (t, *J* = 2.2 Hz).

^{19}F NMR (282 MHz, $CDCl_3$), δ: –86.3 (t, *J* = 15.1 Hz).

Calcd for $C_{13}H_{12}F_2O_2$ (238.23): C 65.54, H 5.08. Found: C 65.48, H 5.19.

4,4-Difluoro-5-phenylpent-2-yn-1-yl benzoate (4f)

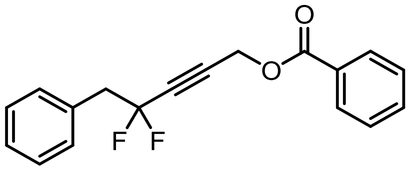

Yield 201 mg (67%). Colorless oil. R_f = 0.21 (Hexane/EtOAc, 15:1).

^{1}H NMR (300 MHz, $CDCl_3$), δ: 3.39 (t, *J* = 14.7 Hz, 2H), 4.95 (t, *J* = 4.1 Hz, 2H), 7.28–7.41 (m, 5H), 7.51 (dd, *J* = 7.6, 7.0 Hz, 2H), 7.64 (t, *J* = 7.6 Hz, 1H), 8.11 (d, *J* = 7.0 Hz, 2H).

^{13}C{^{1}H} NMR (75 MHz, $CDCl_3$), δ: 45.5 (t, *J* = 26.7 Hz), 51.7 (t, *J* = 2.0 Hz), 79.2 (t, *J* = 6.6 Hz), 82.7 (t, *J* = 41.0 Hz), 113.5 (t, *J* = 234.6 Hz), 127.8, 128.4, 128.6, 129.2, 129.9, 130.7, 131.7 (t, *J* = 4.0 Hz), 133.6, 165.5.

^{19}F NMR (282 MHz, $CDCl_3$), δ: –83.9 (tt, *J* = 14.7, 4.1 Hz).

HRMS (ESI): calcd for $C_{18}H_{14}F_2O_2Na$ (M + Na) 323.0854, found 323.0846.

4,4-Difluoro-5-phenylpent-2-yn-1-ol (4g)

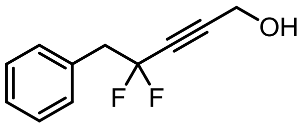

Yield 157 mg (80%). Pale yellow oil. R_f = 0.19 (Hexane/EtOAc, 4:1).

^{1}H NMR (300 MHz, $CDCl_3$), δ: 2.31 (s, 1H), 3.35 (t, *J* = 14.5 Hz, 2H), 4.23 (s, 2H), 7.29–7.39 (m, 5H).

^{13}C{^{1}H} NMR (75 MHz, $CDCl_3$), δ: 45.6 (t, *J* = 27.1 Hz), 50.5 (t, *J* = 1.9 Hz), 78.5 (t, *J* = 40.5 Hz), 86.6 (t, *J* = 6.6 Hz), 113.7 (t, *J* = 234.1 Hz), 127.9, 128.5, 130.7, 132.0 (t, *J* = 4.0 Hz).

^{19}F NMR (282 MHz, $CDCl_3$), δ: –83.1 (t, *J* = 14.5 Hz).

Calcd for $C_{11}H_{10}F_2O$ (196.19): C 67.34, H 5.14. Found: C 67.26, H 5.01.

5,5-Difluoro-6-phenylhex-3-yn-1-ol (4h)

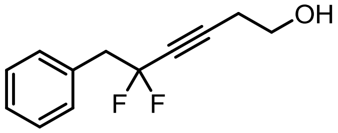

Yield 158 mg (75%). Pale yellow oil. R_f = 0.20 (Hexane/EtOAc, 4:1).

^{1}H NMR (300 MHz, $CDCl_3$), δ: 2.45 (tt, *J* = 5.9, 5.0, 2H), 2.46 (s, 1H), 3.35 (t, *J* = 14.3 Hz, 2H), 3.64 (t, *J* = 5.9 Hz, 2H), 7.28–7.43 (m, 5H).

^{13}C{^{1}H} NMR (75 MHz, $CDCl_3$), δ: 22.6 (t, *J* = 2.0 Hz), 45.7 (t, *J* = 27.5 Hz), 60.1 (t, *J* = 1.9 Hz), 75.1 (t, *J* = 40.1 Hz), 87.1 (t, *J* = 6.6 Hz), 113.9 (t, *J* = 233.2 Hz), 127.8, 128.4, 130.6, 132.4 (t, *J* = 4.1 Hz).

^{19}F NMR (282 MHz, $CDCl_3$), δ: –81.7 (tt, *J* = 14.3, 5.0 Hz).

Calcd for $C_{12}H_{12}F_2O$ (210.22): C 68.56, H 5.75. Found: C 68.77, H 5.71.

Methyl 4-(2,2-difluoro-4-phenylbut-3-yn-1-yl)benzoate (4i)

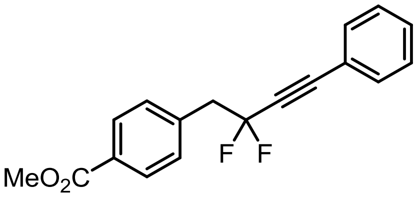

Yield 240 mg (80%). Colorless crystals. Mp 70–71 °C. R_f = 0.24 (Hexane/EtOAc, 10:1).

^{1}H NMR (300 MHz, $CDCl_3$), δ: 3.49 (t, *J* = 14.0 Hz, 2H), 3.92 (s, 3H), 7.28–7.50 (m, 7H), 8.04 (d, 2H, *J* = 8.2 Hz).

^{13}C{^{1}H} NMR (75 MHz, CDCl3), δ: 45.9 (t, *J* = 27.8 Hz), 52.2, 81.2 (t, *J* = 40.2 Hz), 88.5 (t, *J* = 6.9 Hz), 113.9 (t, *J* = 234.3 Hz), 120.0 (t, *J* = 2.6 Hz), 128.6, 129.7, 129.9, 130.1, 130.8, 132.1 (t, *J* = 2.3 Hz), 137.4 (t, *J* = 4.0 Hz), 166.9.

^{19}F NMR (282 MHz, $CDCl_3$), δ: –82.2 (t, *J* = 14.0 Hz).

HRMS (ESI): calcd for $C_{18}H_{14}F_2O_2Na$ (M + Na) 323.0854, found 323.0847.

Diethyl (3,3-difluoro-5-phenylpent-4-yn-1-yl)phosphonate (4j)

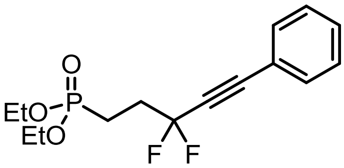

Yield 253 mg (81%). Pale yellow oil. R_f = 0.14 (Hexane/EtOAc, 1:1).

^{1}H NMR (300 MHz, $CDCl_3$), δ: 1.34 (t, *J* = 7.1 Hz, 6H), 1.97–2.12 (m, 2H), 2.32–2.52 (m, 2H), 4.06–4.22 (m, 4H), 7.31–7.52 (m, 5H).

^{13}C{^{1}H} NMR (75 MHz, $CDCl_3$), δ: 16.5 (d, *J* = 5.9 Hz), 19.8 (dt, *J* = 145.8, 3.3 Hz), 33.3 (td, *J* = 28.7, 3.5 Hz), 62.2 (d, *J* = 6.5 Hz), 80.8 (t, *J* = 40.5 Hz), 87.7 (t, *J* = 5.6 Hz), 114.5 (td, *J* = 233.2, 23.6 Hz), 120.0 (t, *J* = 2.6 Hz), 128.6, 130.2, 132.3 (t, *J* = 2.0 Hz).

^{19}F NMR (282 MHz, $CDCl_3$), δ: –85.0 (t, *J* = 13.9 Hz).

HRMS (ESI): calcd for $C_{15}H_{19}F_2O_3PNa$ (M + Na) 339.0932, found 339.0938.

4,4-Difluoro-6-phenylhex-5-yn-1-yl benzoate (4k)

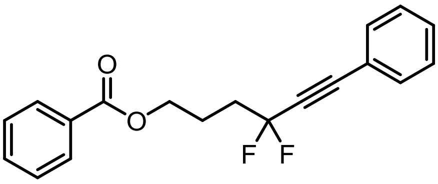

Yield 226 mg (72%). Colorless oil. R_f = 0.21 (Hexane/EtOAc, 15:1).

^{1}H NMR (300 MHz, $CDCl_3$), δ: 2.09–2.22 (m, 2H), 2.27–2.46 (m, 2H), 4.45 (t, *J* = 6.2 Hz, 2H), 7.30–7.61 (m, 8H), 8.08 (d, *J* = 7.3 Hz, 2H).

^{13}C{^{1}H} NMR (75 MHz, $CDCl_3$), δ: 22.8 (t, *J* = 3.4 Hz), 36.4 (t, *J* = 27.3 Hz), 63.8, 81.5 (t, *J* = 40.4 Hz), 87.2 (t, *J* = 6.9 Hz), 115.1 (t, *J* = 232.6 Hz), 120.1 (t, *J* = 2.9 Hz), 128.5, 128.6, 129.8, 130.0, 130.3, 132.2 (t, *J* = 2.3 Hz), 133.1, 166.5.

^{19}F NMR (282 MHz, $CDCl_3$), δ: –83.0 (t, *J* = 14.4 Hz).

Calcd for $C_{19}H_{16}F_2O_2$ (314.33): C 72.60, H 5.13. Found: C 72.41, H 5.19.

4,4-Difluoro-7-hydroxyhept-5-yn-1-yl benzoate (4l)

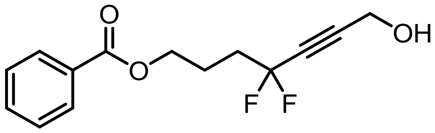

Yield 190 mg (71%). Pale yellow oil. R_f = 0.28 (Hexane/EtOAc, 2:1).

^{1}H NMR (300 MHz, $CDCl_3$), δ: 1.98–2.12 (m, 2H), 2.13–2.32 (m, 2H), 2.61 (t, *J* = 6.2 Hz, 1H), 4.31–4.43 (m, 4H), 7.45 (t, *J* = 7.5 Hz, 2H), 7.57 (t, *J* = 7.4 Hz, 1H), 8.03 (dd, *J* = 7.5, 7.4 Hz, 2H).

^{13}C{^{1}H} NMR (75 MHz, $CDCl_3$), δ: 22.5 (t, *J* = 3.8 Hz), 36.0 (t, *J* = 26.9 Hz), 50.5 (t, *J* = 1.7 Hz), 63.8, 78.3 (t, *J* = 40.7 Hz), 86.0 (t, *J* = 6.6 Hz), 114.4 (t, *J* = 232.7 Hz), 128.6, 129.7, 130.0, 133.2, 166.8.

^{19}F NMR (282 MHz, $CDCl_3$), δ: –83.8 (t, *J* = 14.3 Hz).

HRMS (ESI): calcd for $C_{14}H_{14}F_2O_3Na$ (M + Na) 291.0803, found 291.0801.

4,4-Difluoro-7-hydroxy-3-methylhept-5-yn-1-yl benzoate (4m)

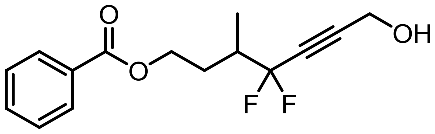

Yield 175 mg (62%). Pale yellow oil. R_f = 0.18 (Hexane/EtOAc, 4:1).

^{1}H NMR (300 MHz, $CDCl_3$), δ: 1.16 (d, *J* = 6.9 Hz, 3H), 1.71 (qt, *J* = 6.1, 5.8 Hz, 1H), 2.14–2.37 (m, 2H), 2.80 (s, 1H), 4.31–4.46 (m, 4H), 7.43 (dd, *J* = 8.3, 7.1 Hz, 2H), 7.56 (tt, J = 7.1, 1.2 Hz, 1H), 8.02 (dd, *J* = 8.3, 1.2 Hz, 2H).

^{13}C{^{1}H} NMR (75 MHz, $CDCl_3$), δ: 13.5 (t, *J* = 4.0 Hz), 29.6 (t, *J* = 2.9 Hz), 39.1 (t, *J* = 25.0 Hz), 50.4 (t, *J* = 2.0 Hz), 62.7, 77.3 (m), 86.7 (t, *J* = 6.9 Hz), 116.8 (t, *J* = 235.2 Hz), 128.5, 129.7, 130.1, 133.2, 166.9.

^{19}F NMR (282 MHz, $CDCl_3$), δ: –89.7 (m).

HRMS (ESI): calcd for $C_{15}H_{16}F_2O_3Na$ (M + Na) 305.0960, found 305.0954.

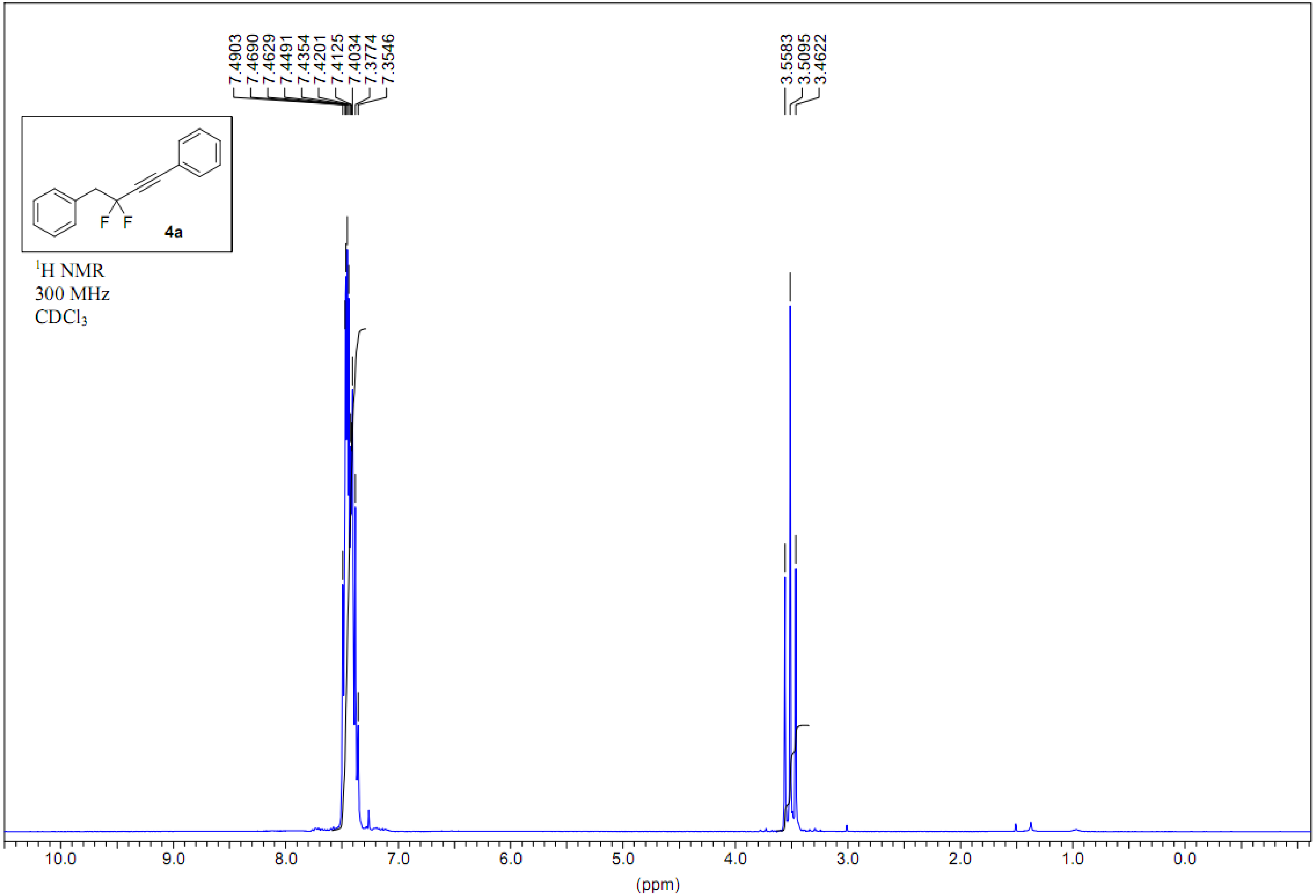

^{1}H NMR
300 MHz
$CDCl_3$

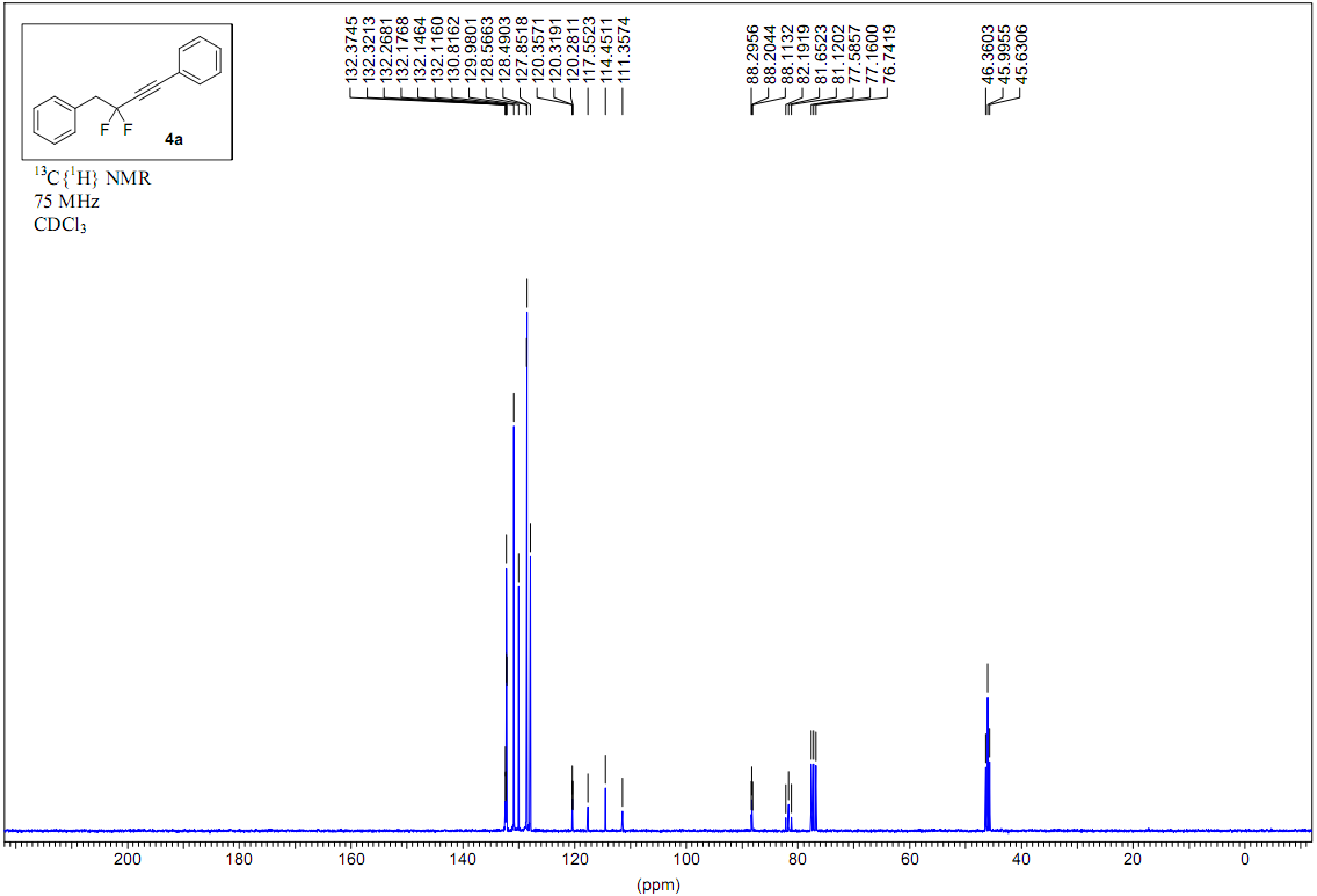

4a
$^{13}C\{^1H\}$ NMR
75 MHz
$CDCl_3$
132.3745
132.3213
132.2681
132.1768
132.1464
132.1160
130.8162
129.9801
128.5663
128.4903
127.8518
120.3571
120.3191
120.2811
117.5523
114.4511
111.3574
88.2956
88.2044
88.1132
82.1919
81.6523
81.1202
77.5857
77.1600
76.7419
46.3603
45.9955
45.6306
200
180
160
140
120
100
80
60
40
20
0
(ppm)

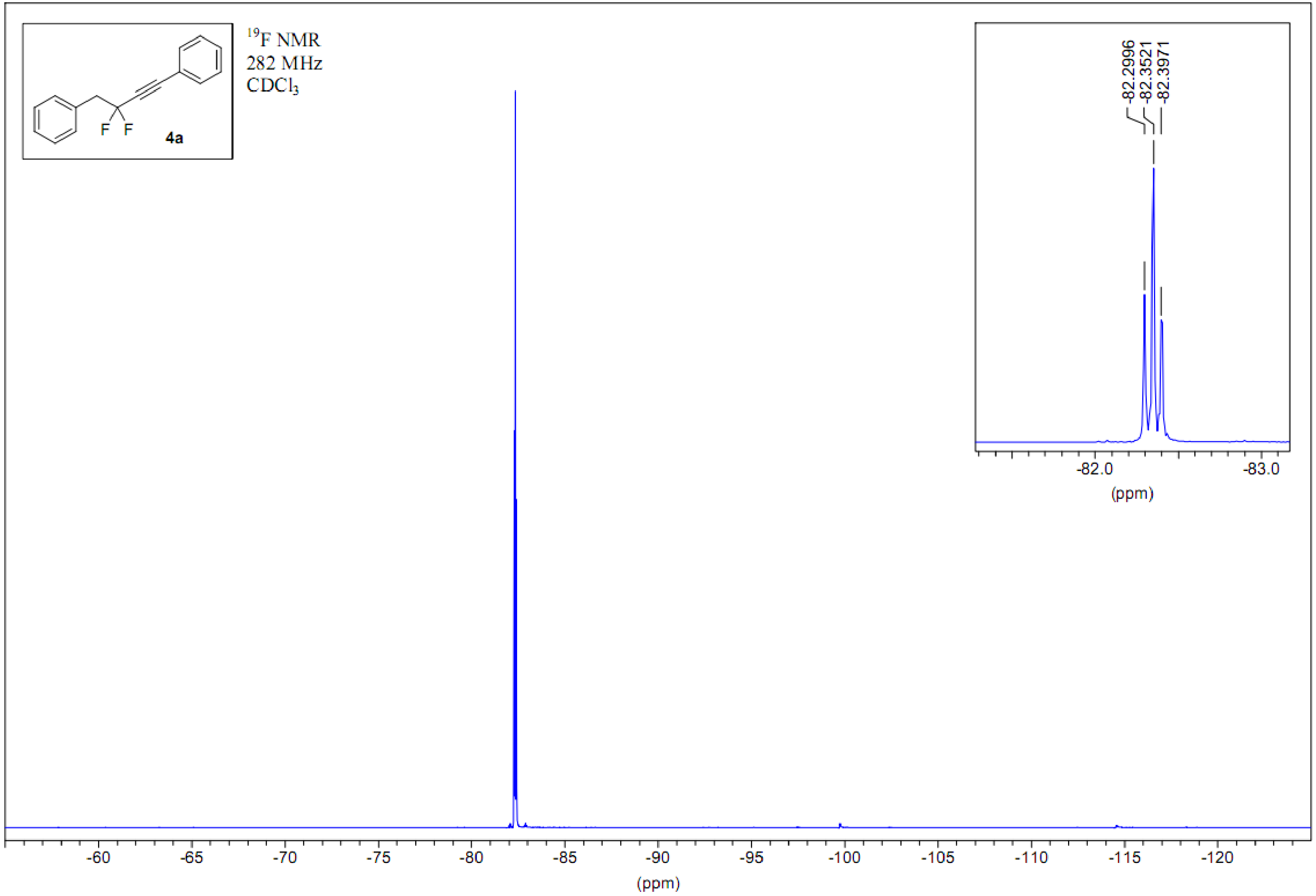
4a
19F NMR
282 MHz
CDCl3
-82.2996
-82.3521
-82.3971
-82.0
-83.0
(ppm)
-60
-65
-70
-75
-80
-85
-90
-95
-100
-105
-110
-115
-120
(ppm)

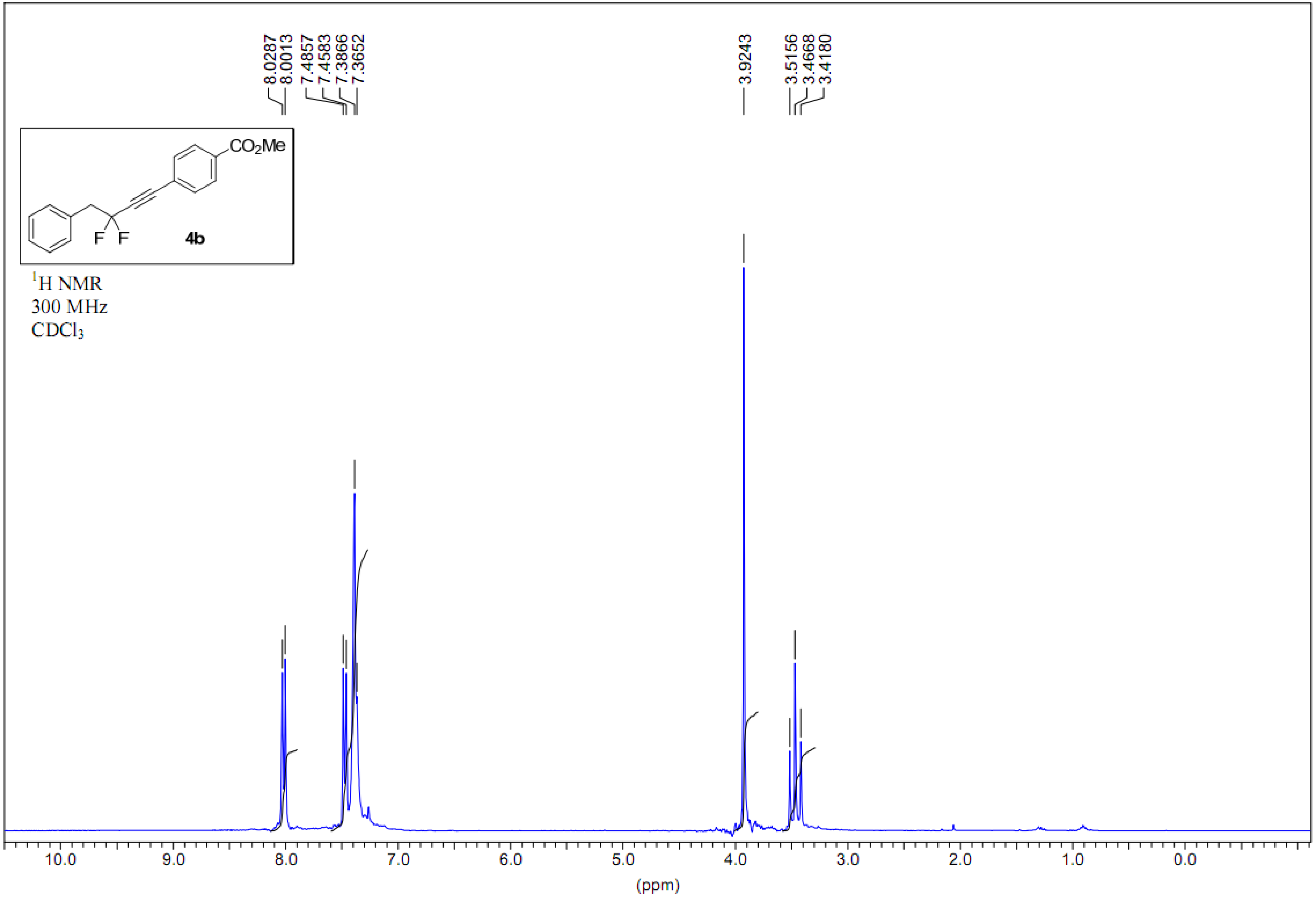
8.0287
8.0013
7.4857
7.4583
7.3866
7.3652
3.9243
3.5156
3.4668
3.4180
CO2Me
F F
4b
1H NMR
300 MHz
CDCl3
10.0 9.0 8.0 7.0 6.0 5.0 4.0 3.0 2.0 1.0 0.0
(ppm)

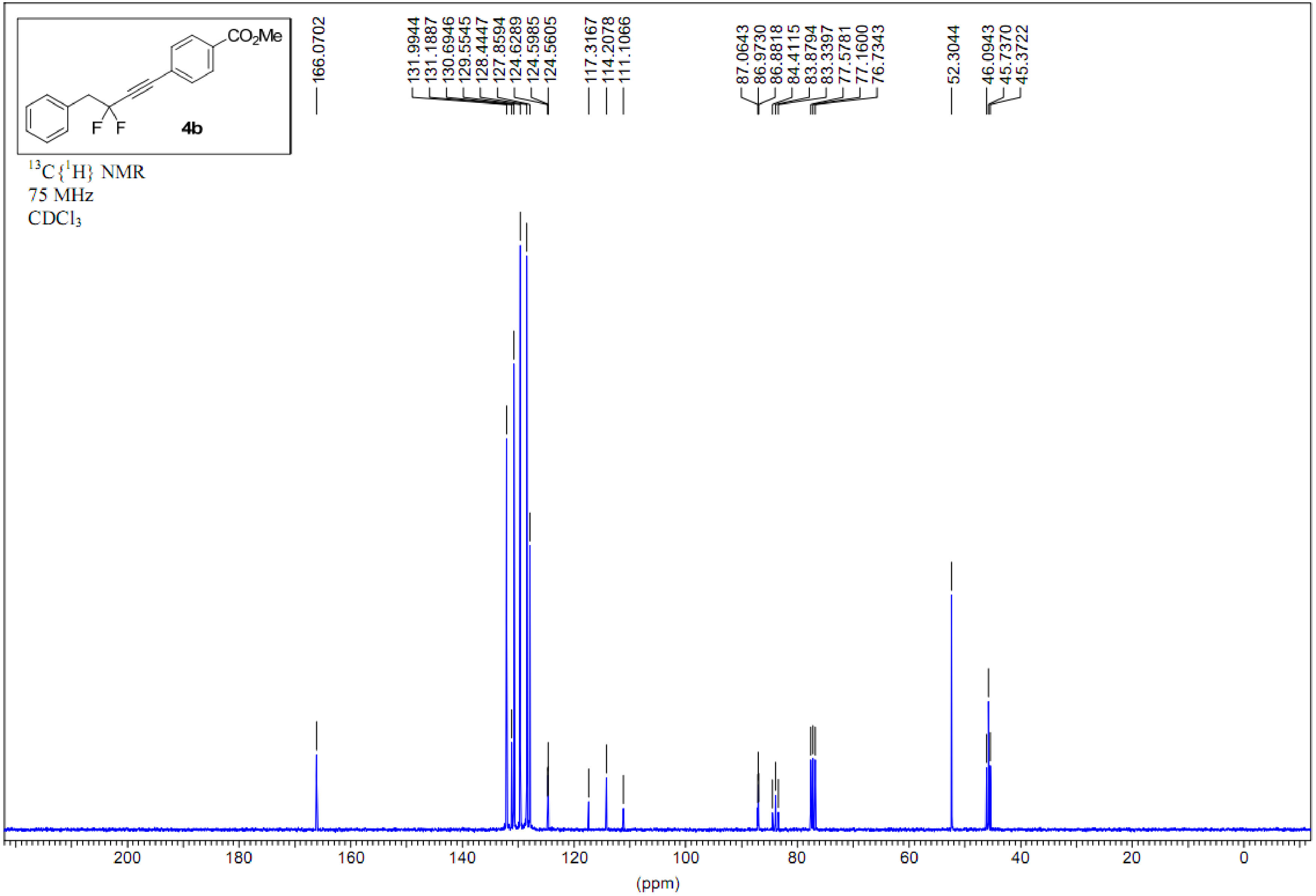
CO2Me
F F
4b
13C{1H} NMR
75 MHz
CDCl3
166.0702
131.9944
131.1887
130.6946
129.5545
128.4447
127.8594
124.6289
124.5985
124.5605
117.3167
114.2078
111.1066
87.0643
86.9730
86.8818
84.4115
83.8794
83.3397
77.5781
77.1600
76.7343
52.3044
46.0943
45.7370
45.3722
200
180
160
140
120
100
80
60
40
20
0
(ppm)

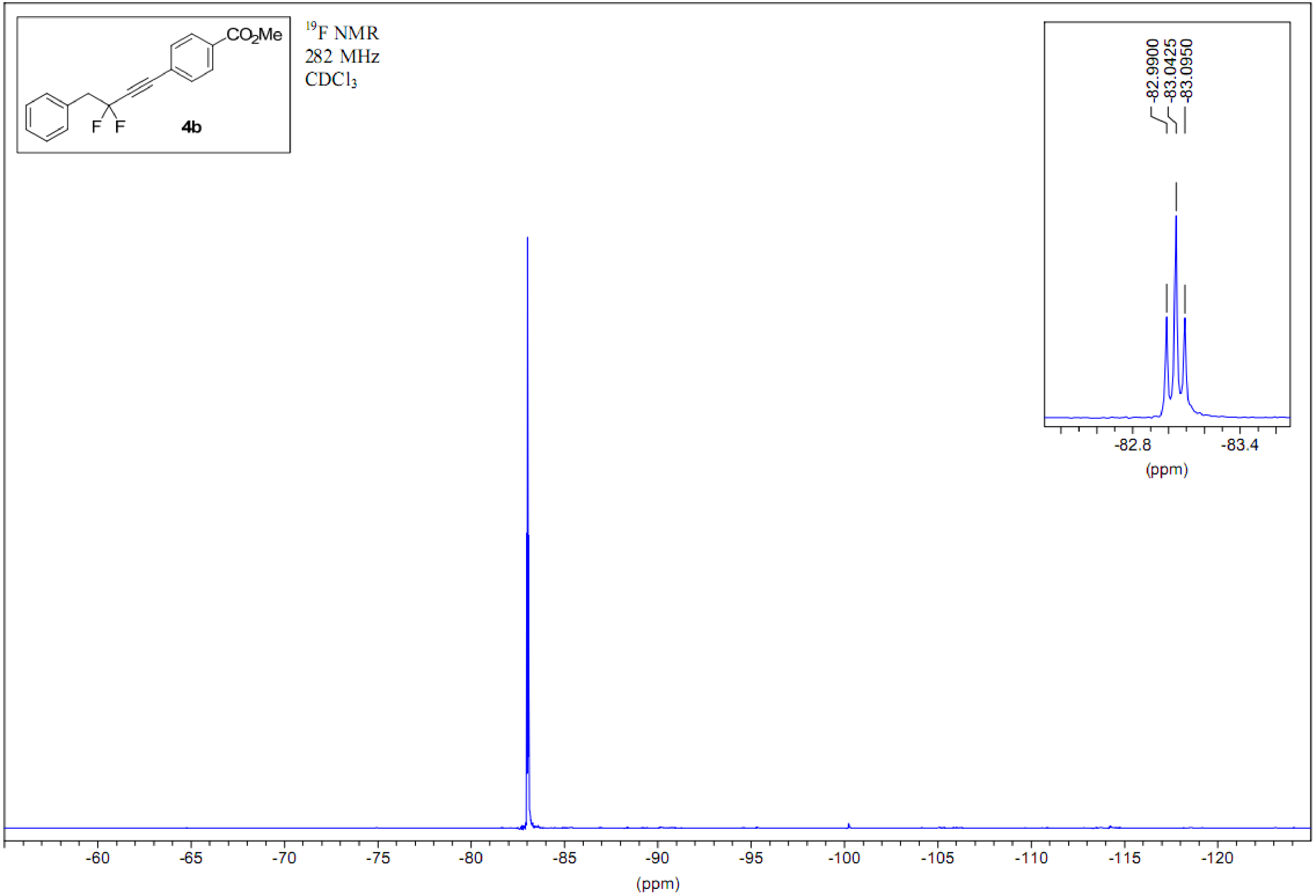
CO2Me
F F
4b
19F NMR
282 MHz
CDCl3
-82.9900
-83.0425
-83.0950
-82.8
-83.4
(ppm)
-60
-65
-70
-75
-80
-85
-90
-95
-100
-105
-110
-115
-120
(ppm)

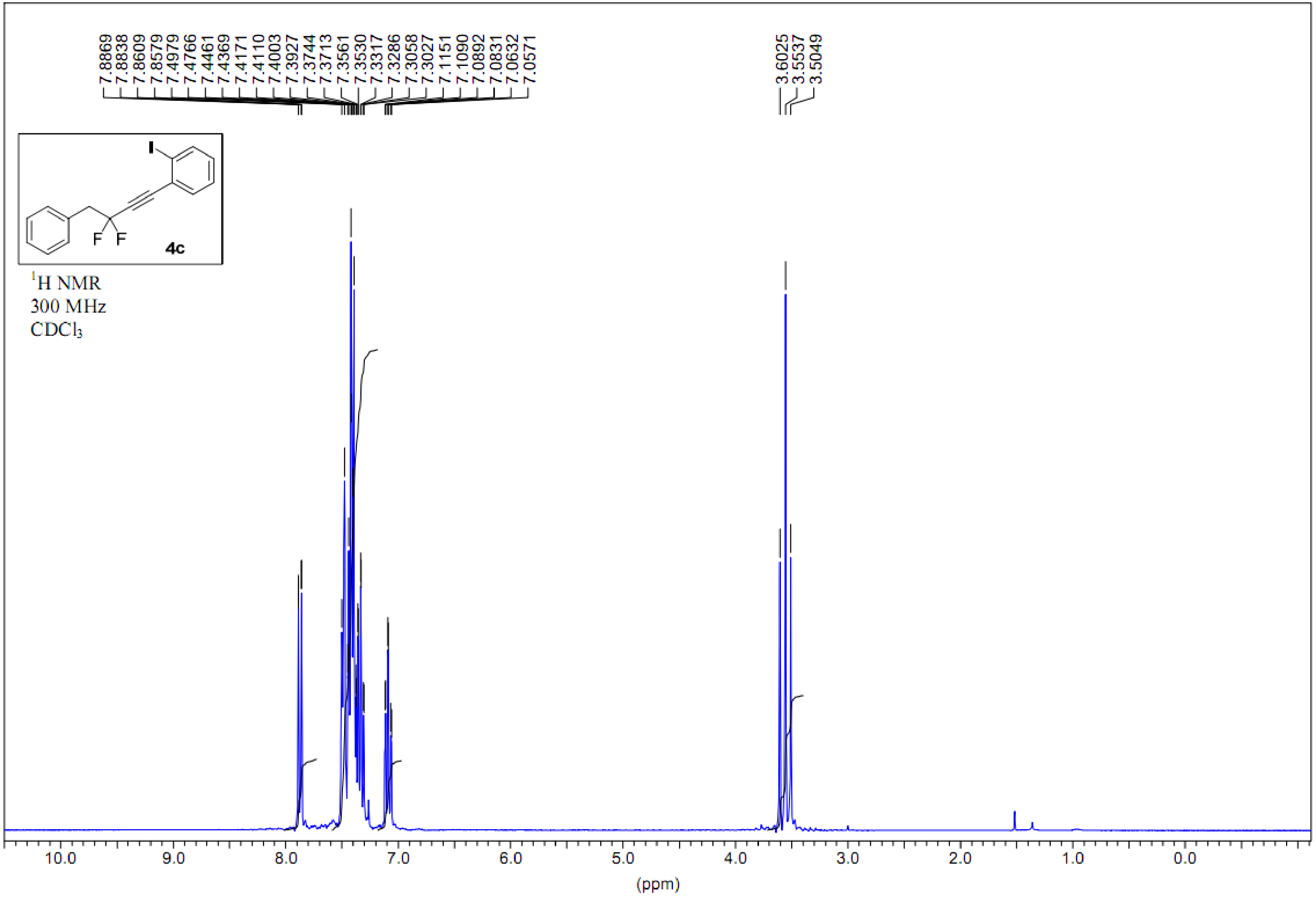
7.8869
7.8838
7.8609
7.8579
7.4979
7.4766
7.4461
7.4369
7.4171
7.4110
7.4003
7.3927
7.3744
7.3713
7.3561
7.3530
7.3317
7.3286
7.3058
7.3027
7.1151
7.1090
7.0892
7.0831
7.0632
7.0571
3.6025
3.5537
3.5049
I
F
F
4c
1H NMR
300 MHz
CDCl3
10.0
9.0
8.0
7.0
6.0
5.0
4.0
3.0
2.0
1.0
0.0
(ppm)

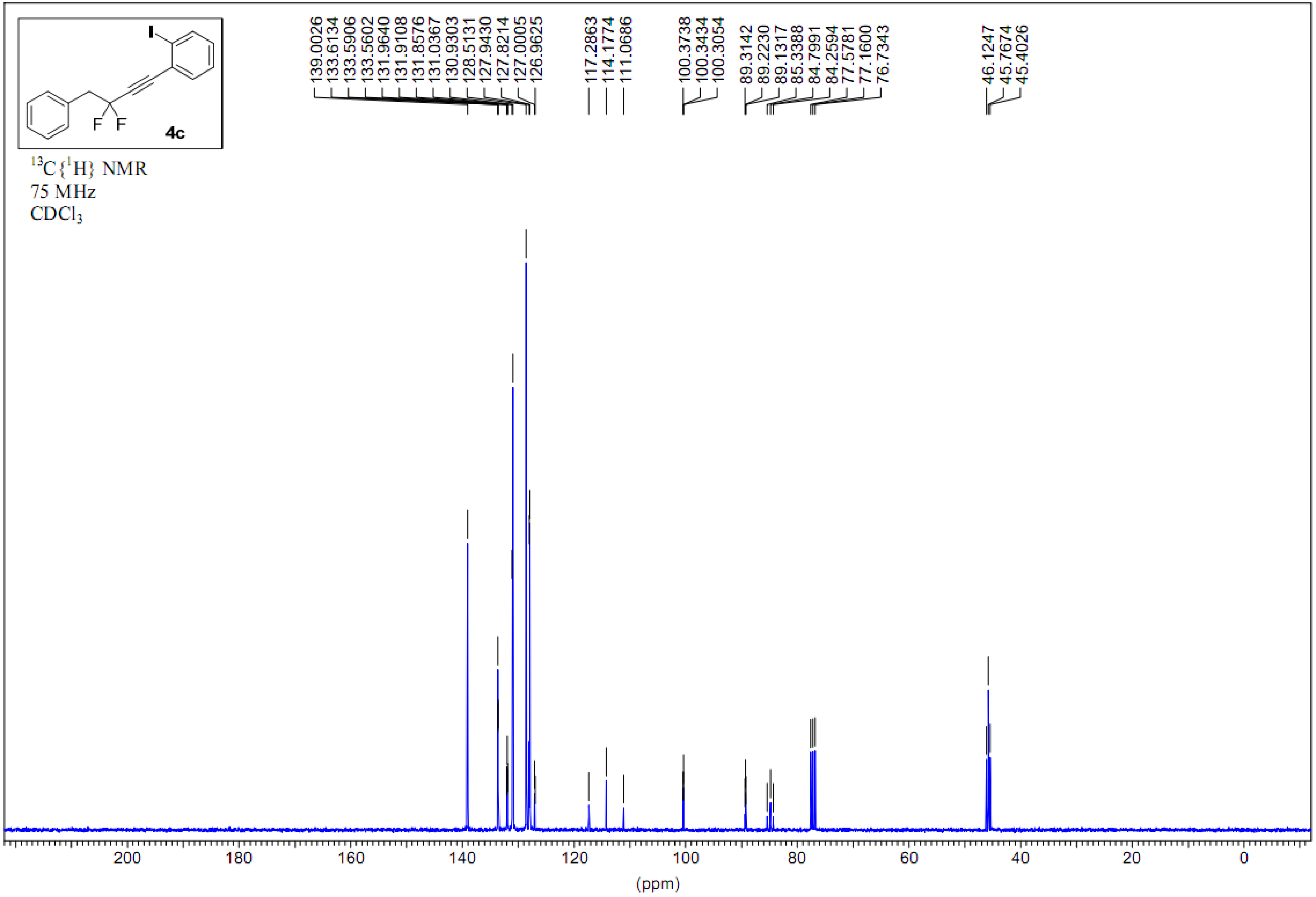

I
F F
4c
$^{13}C\{^{1}H\}$ NMR
75 MHz
$CDCl_3$
139.0026
133.6134
133.5906
133.5602
131.9640
131.9108
131.8576
131.0367
130.9303
128.5131
127.9430
127.8214
127.0005
126.9625
117.2863
114.1774
111.0686
100.3738
100.3434
100.3054
89.3142
89.2230
89.1317
85.3388
84.7991
84.2594
77.5781
77.1600
76.7343
46.1247
45.7674
45.4026
200
180
160
140
120
100
80
60
40
20
0
(ppm)

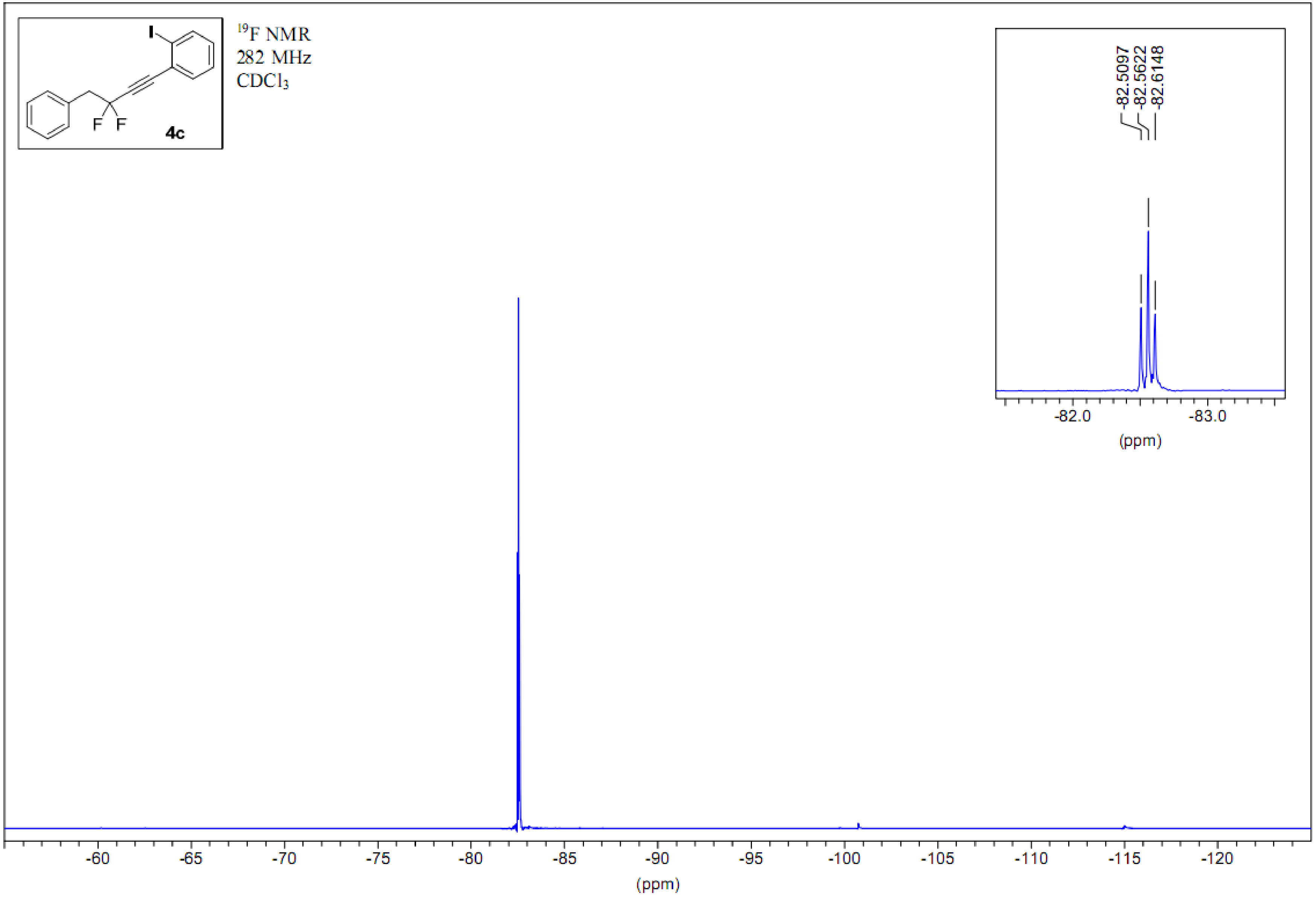

I
F
F
4c
^{19}F NMR
282 MHz
CDCl3
-82.5097
-82.5622
-82.6148
-82.0
-83.0
(ppm)
-60
-65
-70
-75
-80
-85
-90
-95
-100
-105
-110
-115
-120
(ppm)

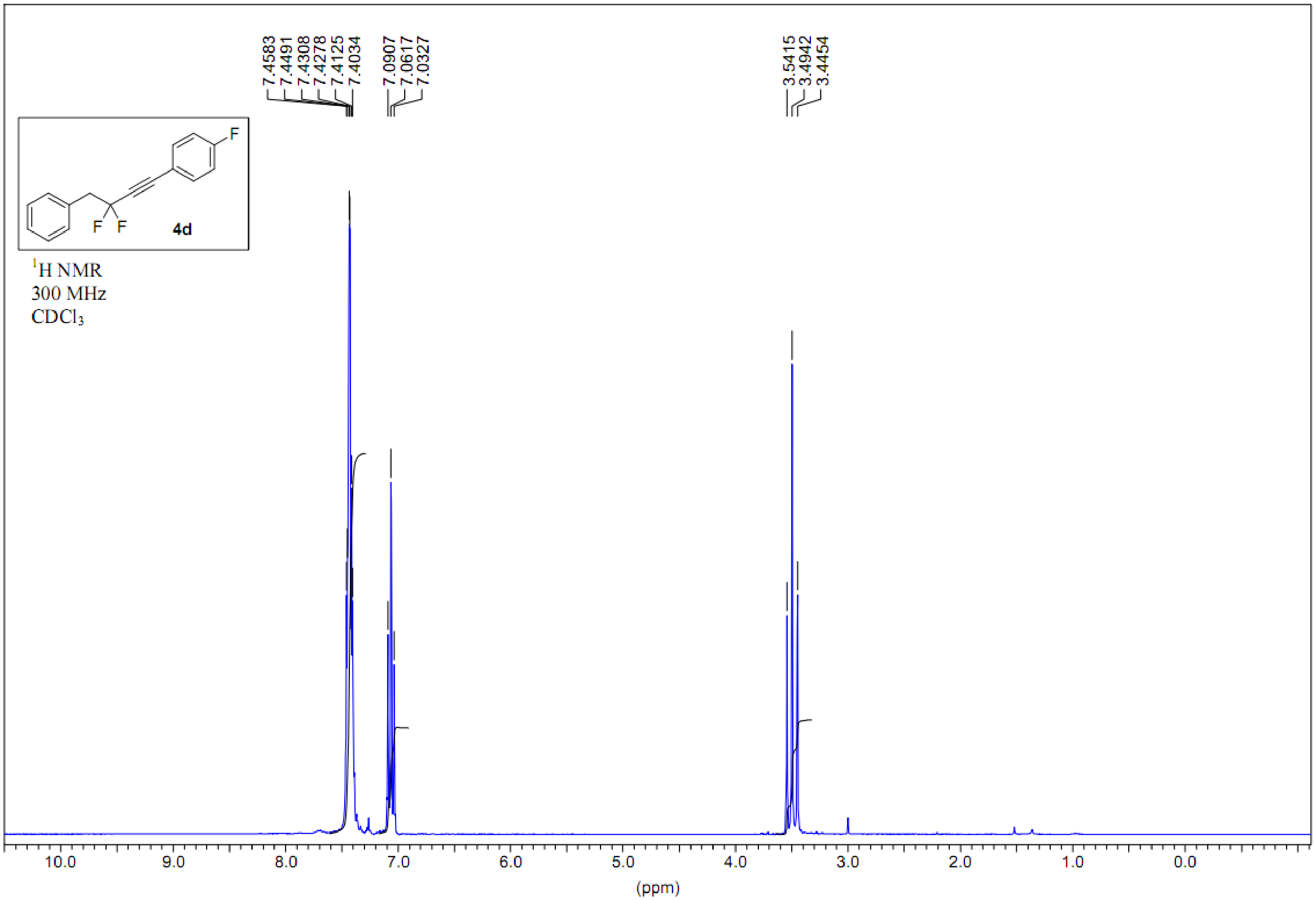
7.4583
7.4491
7.4308
7.4278
7.4125
7.4034
7.0907
7.0617
7.0327
3.5415
3.4942
3.4454
F
F F
4d
1H NMR
300 MHz
CDCl3
10.0
9.0
8.0
7.0
6.0
5.0
4.0
3.0
2.0
1.0
0.0
(ppm)

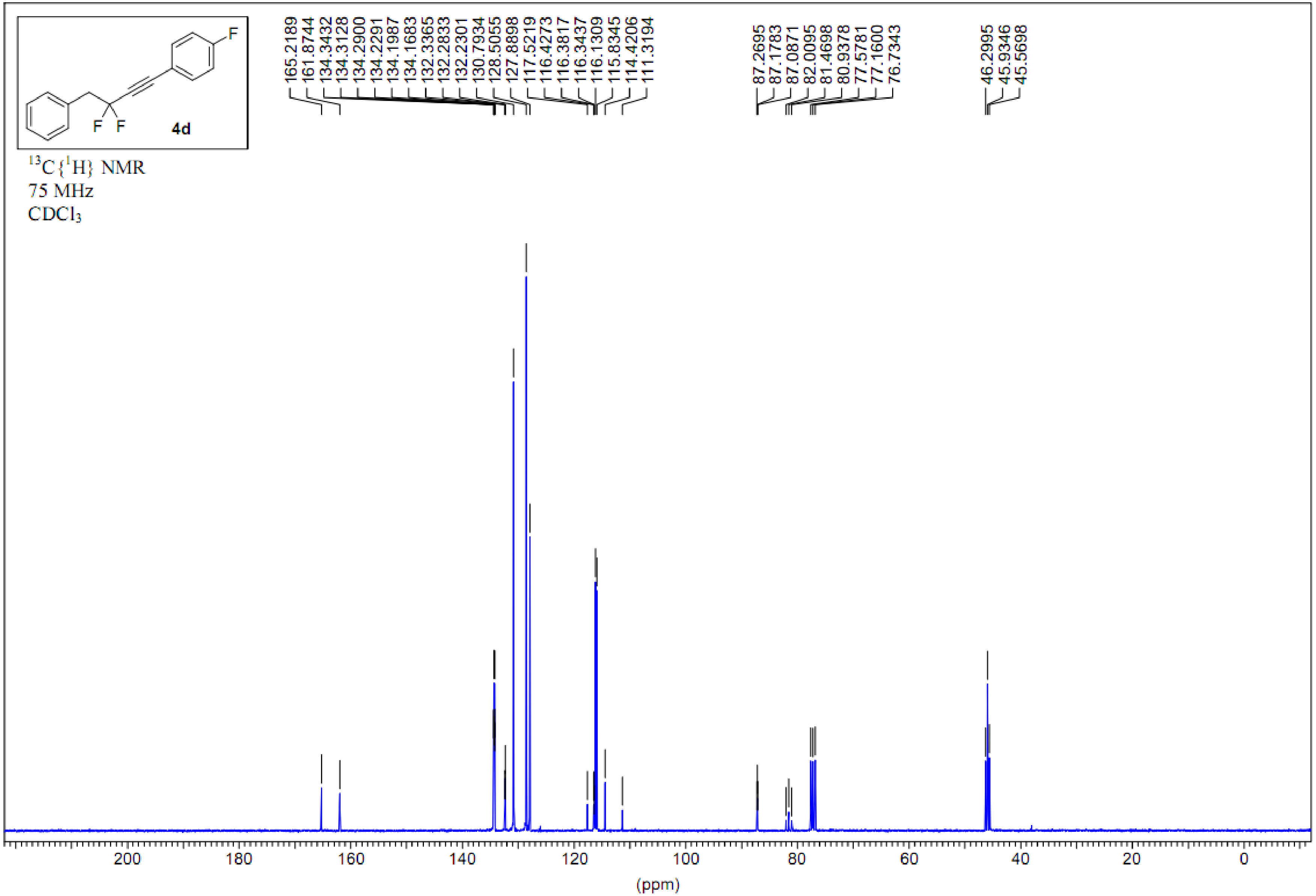

4d
F
F F
$^{13}C\{^1H\}$ NMR
75 MHz
$CDCl_3$
165.2189
161.8744
134.3432
134.3128
134.2900
134.2291
134.1987
134.1683
132.3365
132.2833
132.2301
130.7934
128.5055
127.8898
117.5219
116.4273
116.3817
116.3437
116.1309
115.8345
114.4206
111.3194
87.2695
87.1783
87.0871
82.0095
81.4698
80.9378
77.5781
77.1600
76.7343
46.2995
45.9346
45.5698
200
180
160
140
120
100
80
60
40
20
0
(ppm)

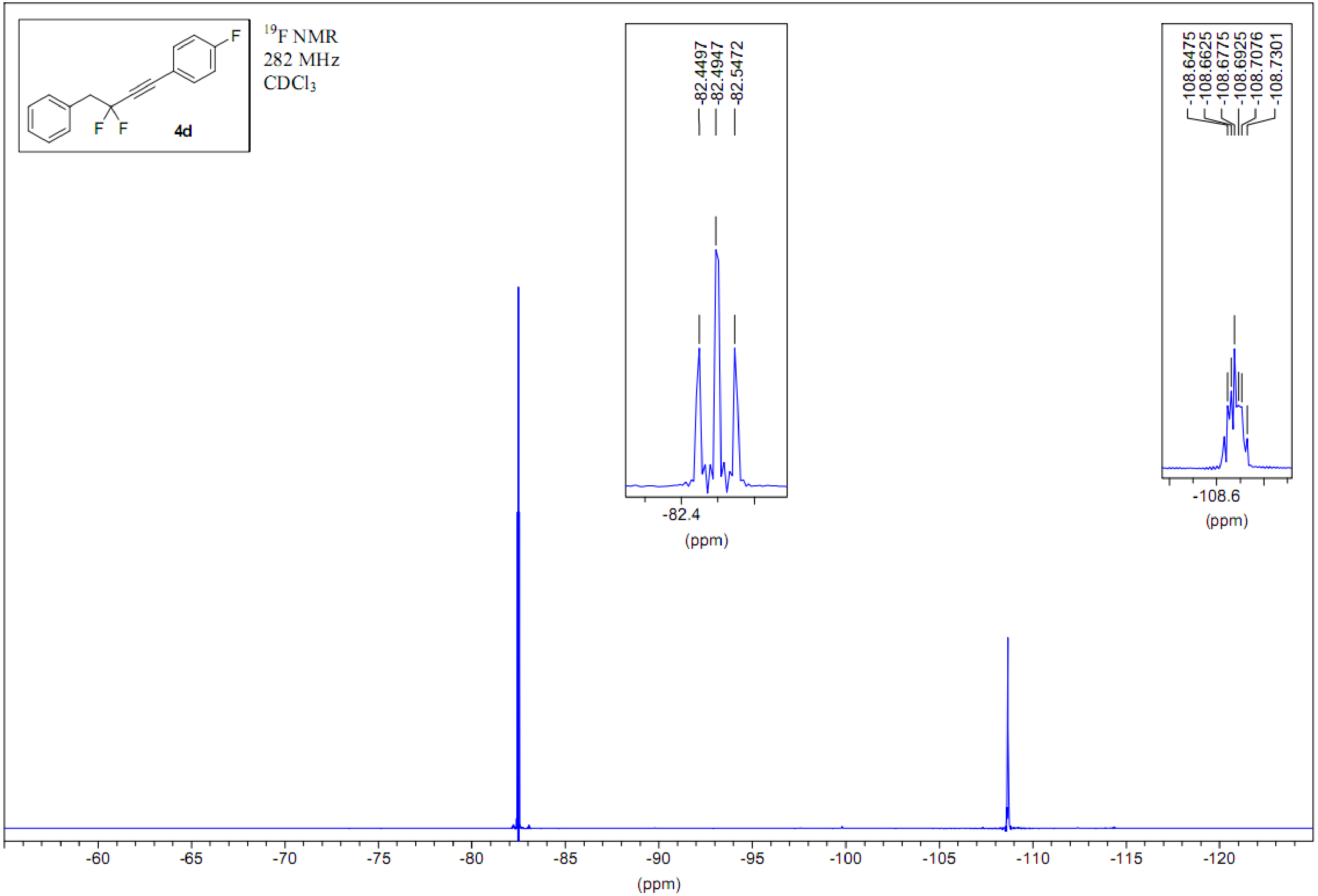

F
F F
4d
19F NMR
282 MHz
CDCl3
-82.4497
-82.4947
-82.5472
-82.4
(ppm)
-108.6475
-108.6625
-108.6775
-108.6925
-108.7076
-108.7301
-108.6
(ppm)
-60
-65
-70
-75
-80
-85
-90
-95
-100
-105
-110
-115
-120
(ppm)

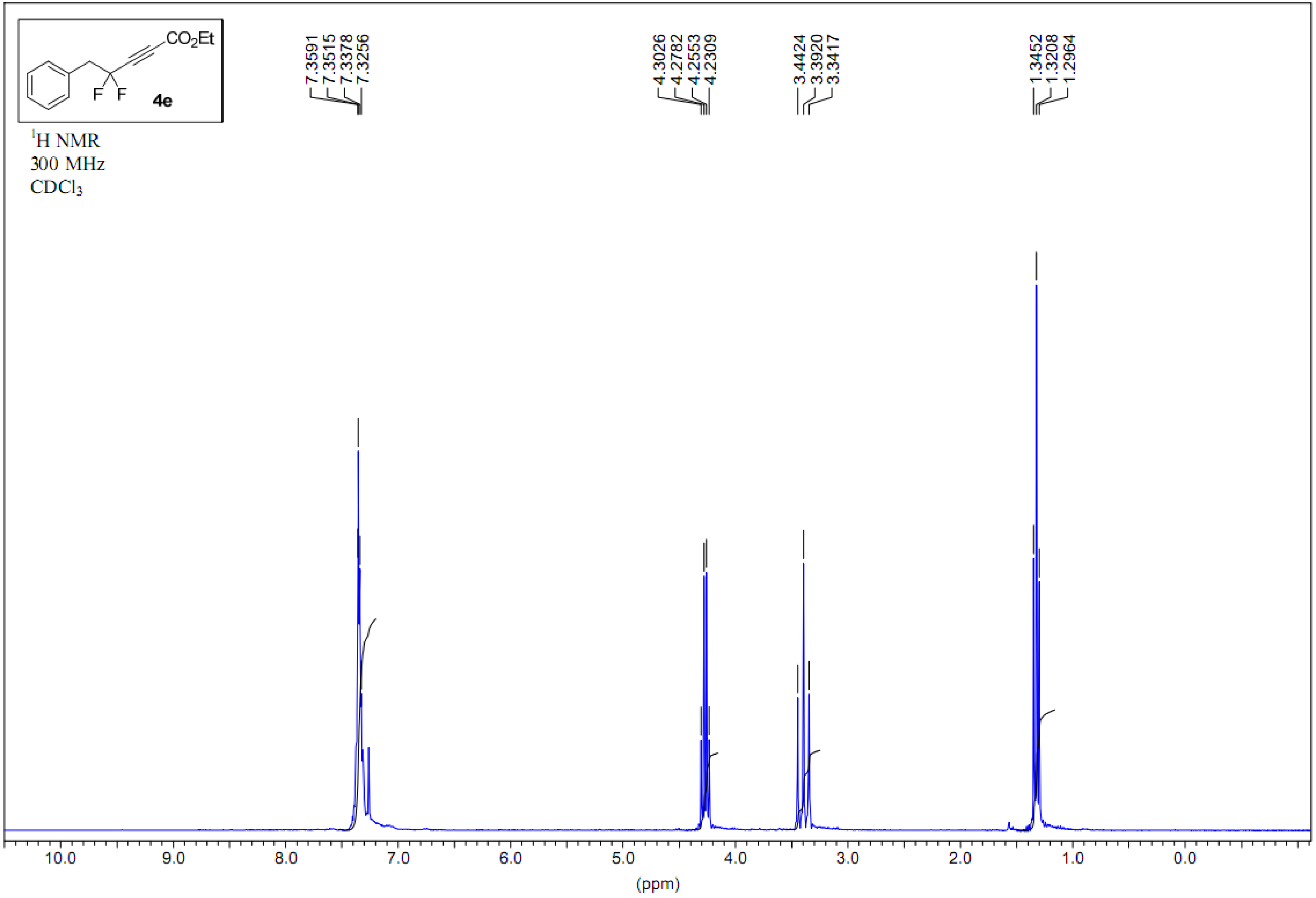
CO2Et
F F
4e
1H NMR
300 MHz
CDCl3
7.3591
7.3515
7.3378
7.3256
4.3026
4.2782
4.2553
4.2309
3.4424
3.3920
3.3417
1.3452
1.3208
1.2964
10.0
9.0
8.0
7.0
6.0
5.0
4.0
3.0
2.0
1.0
0.0
(ppm)

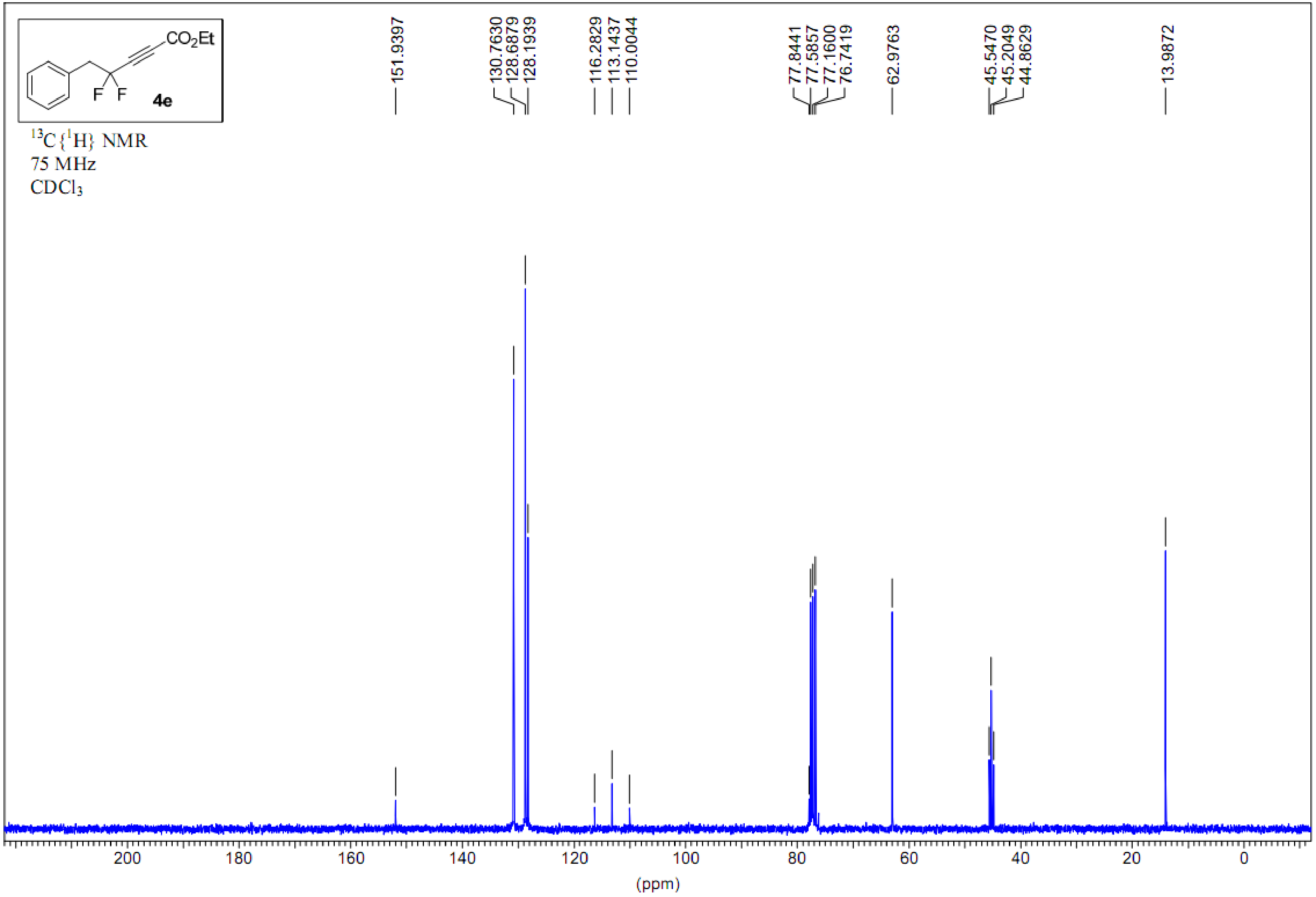
CO2Et
F F
4e
13C {1H} NMR
75 MHz
CDCl3
151.9397
130.7630
128.6879
128.1939
116.2829
113.1437
110.0044
77.8441
77.5857
77.1600
76.7419
62.9763
45.5470
45.2049
44.8629
13.9872
200
180
160
140
120
100
80
60
40
20
0
(ppm)

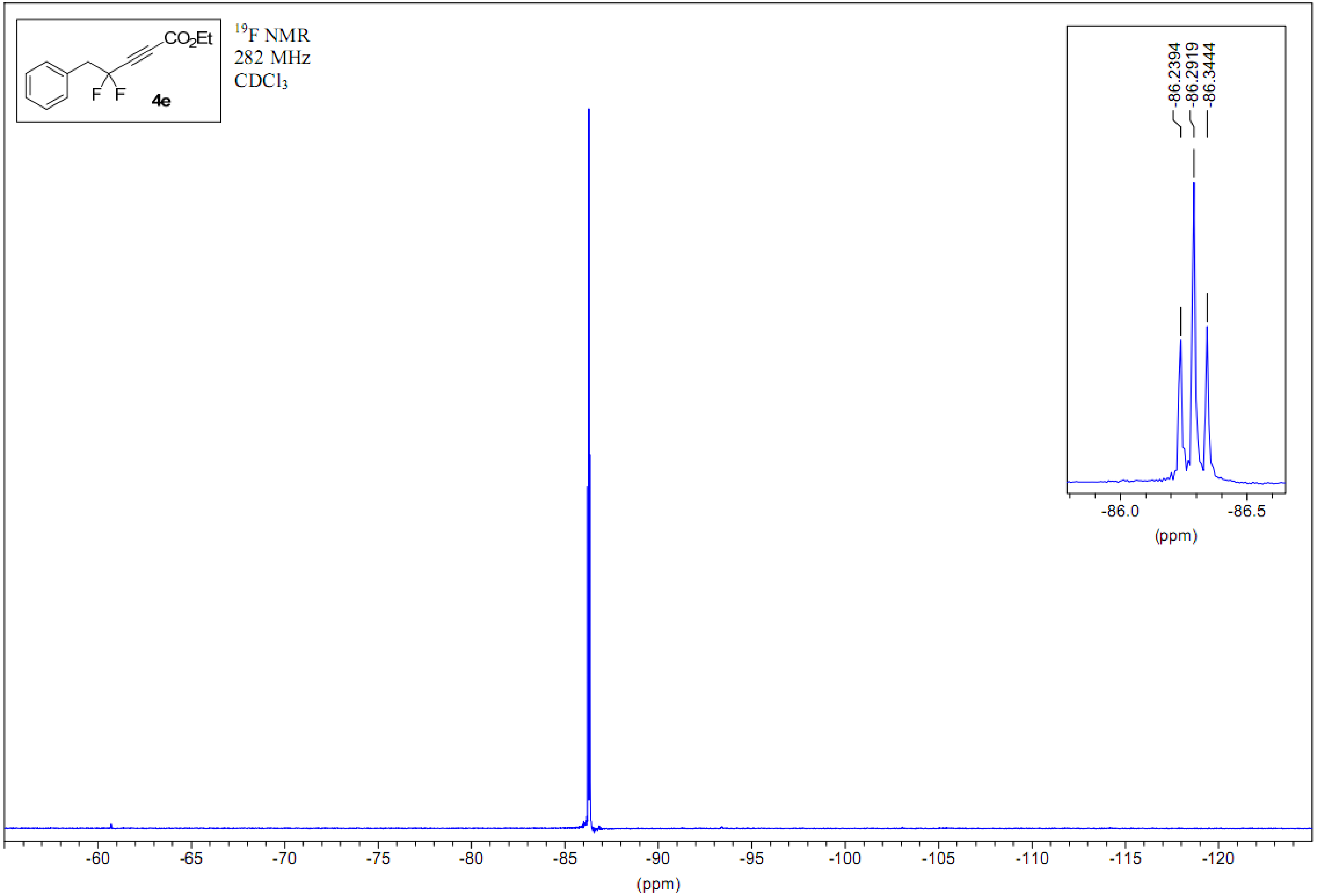

CO_2Et
F F
4e
^{19}F NMR
282 MHz
$CDCl_3$
-86.2394
-86.2919
-86.3444
-86.0
-86.5
(ppm)
-60
-65
-70
-75
-80
-85
-90
-95
-100
-105
-110
-115
-120
(ppm)

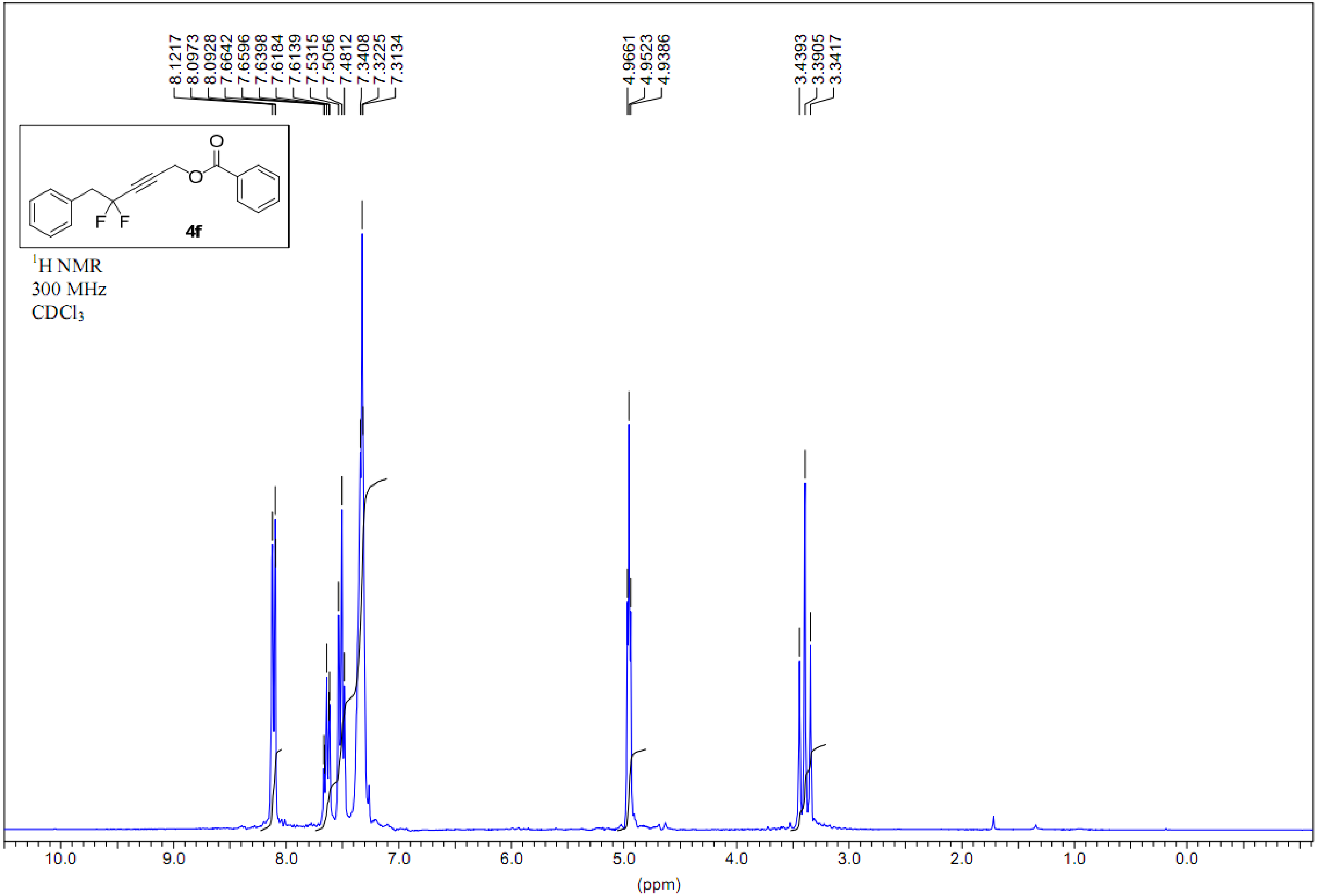

8.1217
8.0973
8.0928
7.6642
7.6596
7.6398
7.6184
7.6139
7.5315
7.5056
7.4812
7.3408
7.3225
7.3134
4.9661
4.9523
4.9386
3.4393
3.3905
3.3417
O
O
F F
4f
1H NMR
300 MHz
CDCl3
10.0
9.0
8.0
7.0
6.0
5.0
4.0
3.0
2.0
1.0
0.0
(ppm)

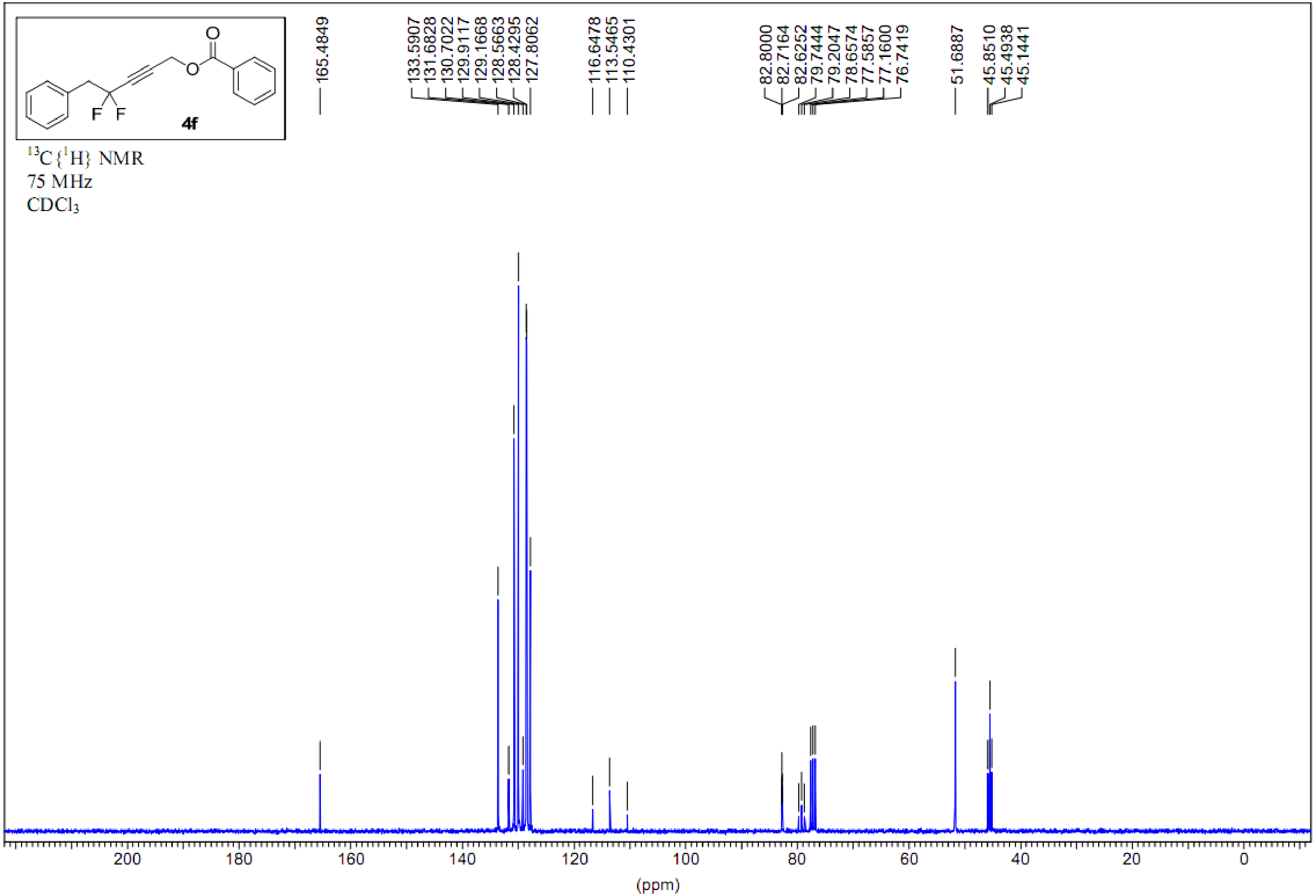

4f
$^{13}C\{^1H\}$ NMR
75 MHz
$CDCl_3$
165.4849
133.5907
131.6828
130.7022
129.9117
129.1668
128.5663
128.4295
127.8062
116.6478
113.5465
110.4301
82.8000
82.7164
82.6252
79.7444
79.2047
78.6574
77.5857
77.1600
76.7419
51.6887
45.8510
45.4938
45.1441
(ppm)

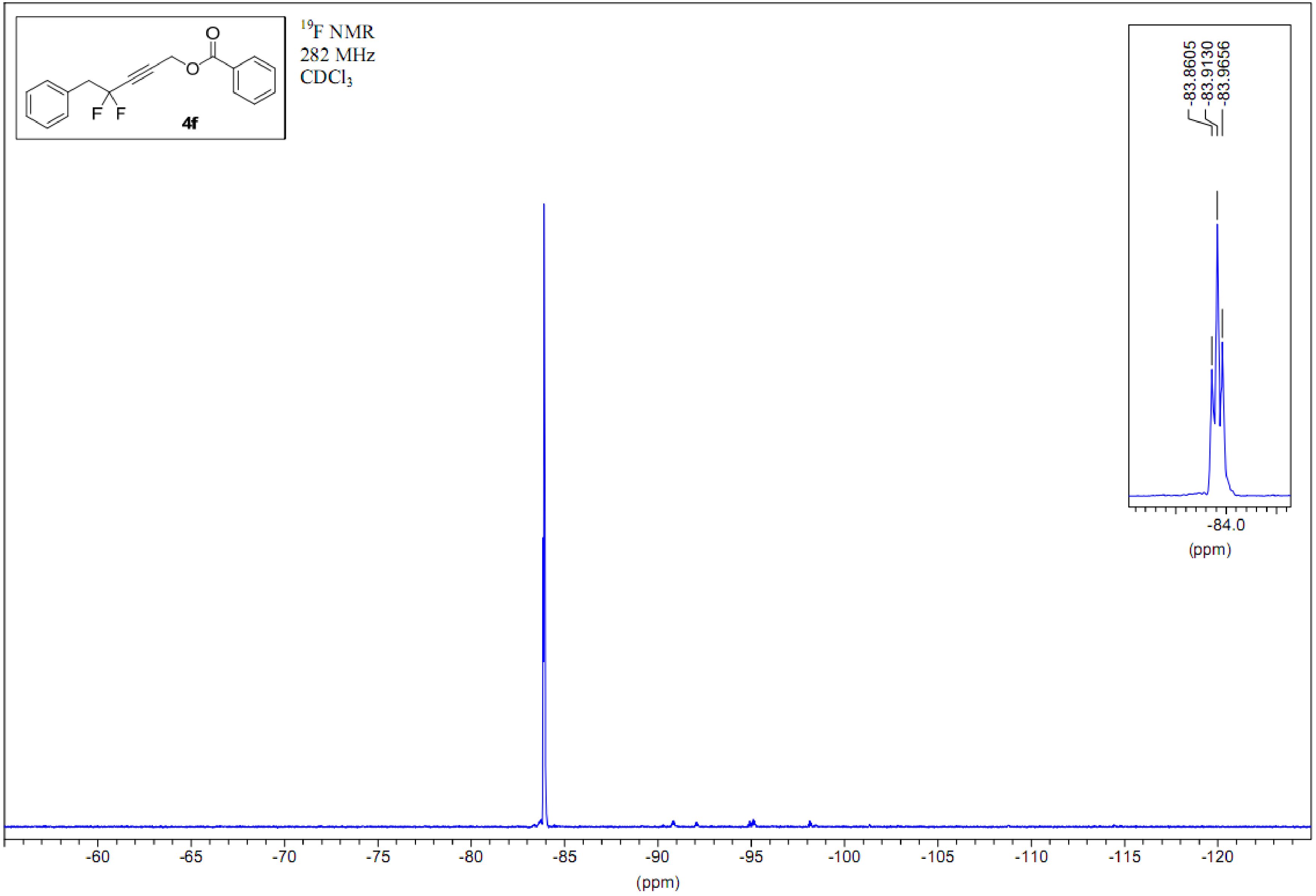

F F
4f
^{19}F NMR
282 MHz
$CDCl_3$
-83.8605
-83.9130
-83.9656
-84.0
(ppm)
-60
-65
-70
-75
-80
-85
-90
-95
-100
-105
-110
-115
-120
(ppm)

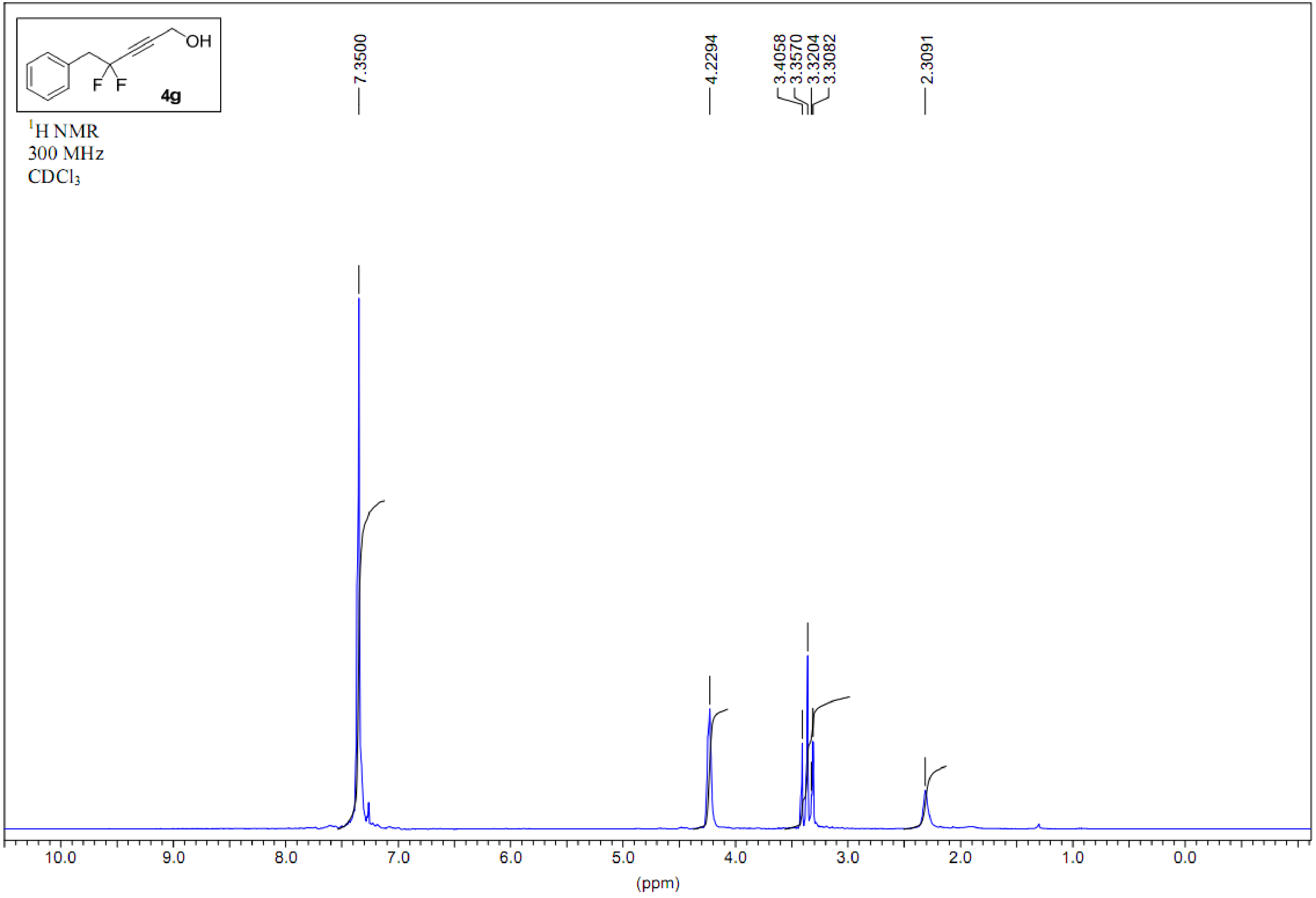

OH
F F
4g
1H NMR
300 MHz
CDCl3
7.3500
4.2294
3.4058
3.3570
3.3204
3.3082
2.3091
10.0
9.0
8.0
7.0
6.0
5.0
4.0
3.0
2.0
1.0
0.0
(ppm)

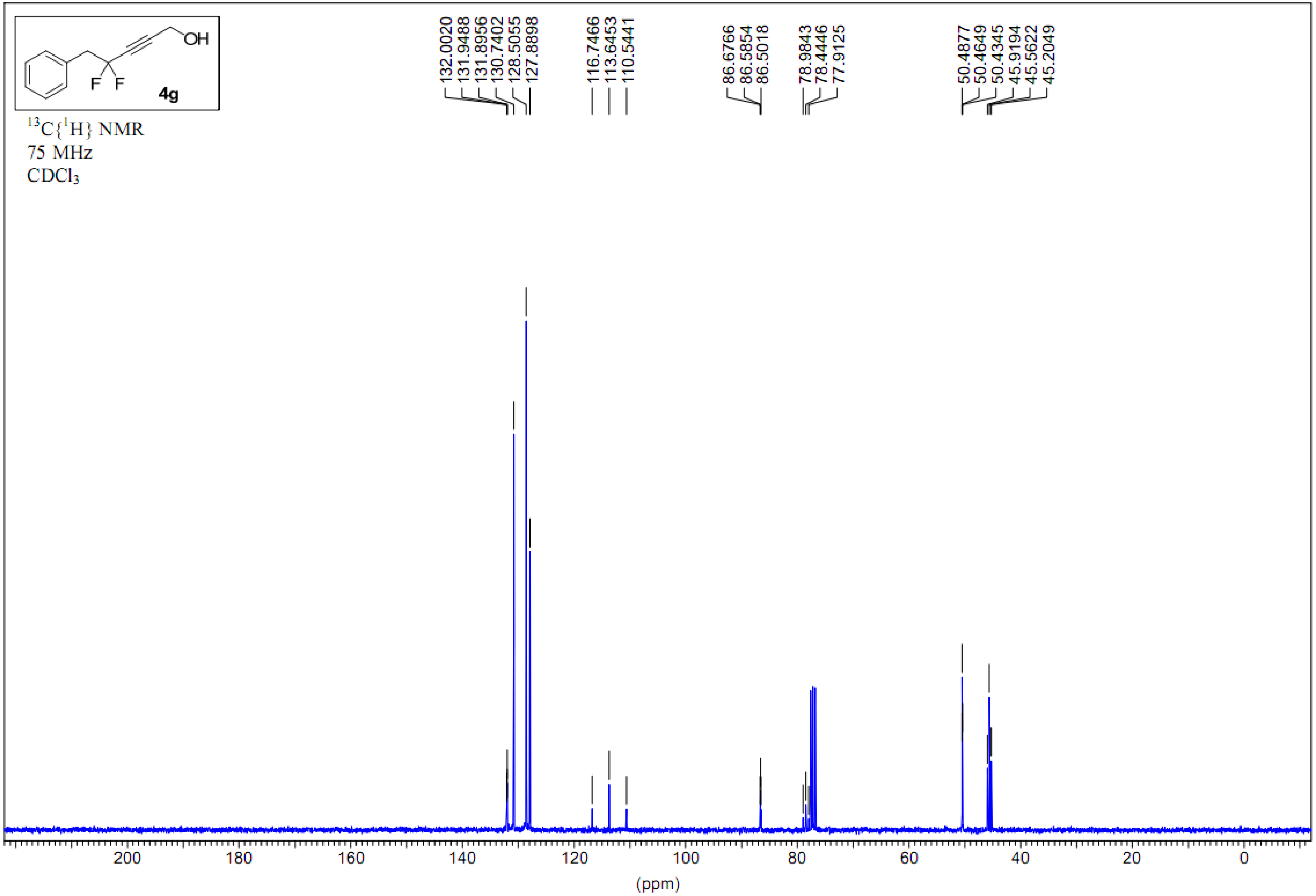
OH
F F
4g
$^{13}C\{^{1}H\}$ NMR
75 MHz
$CDCl_3$
132.0020
131.9488
131.8956
130.7402
128.5055
127.8898
116.7466
113.6453
110.5441
86.6766
86.5854
86.5018
78.9843
78.4446
77.9125
50.4877
50.4649
50.4345
45.9194
45.5622
45.2049
200
180
160
140
120
100
80
60
40
20
0
(ppm)

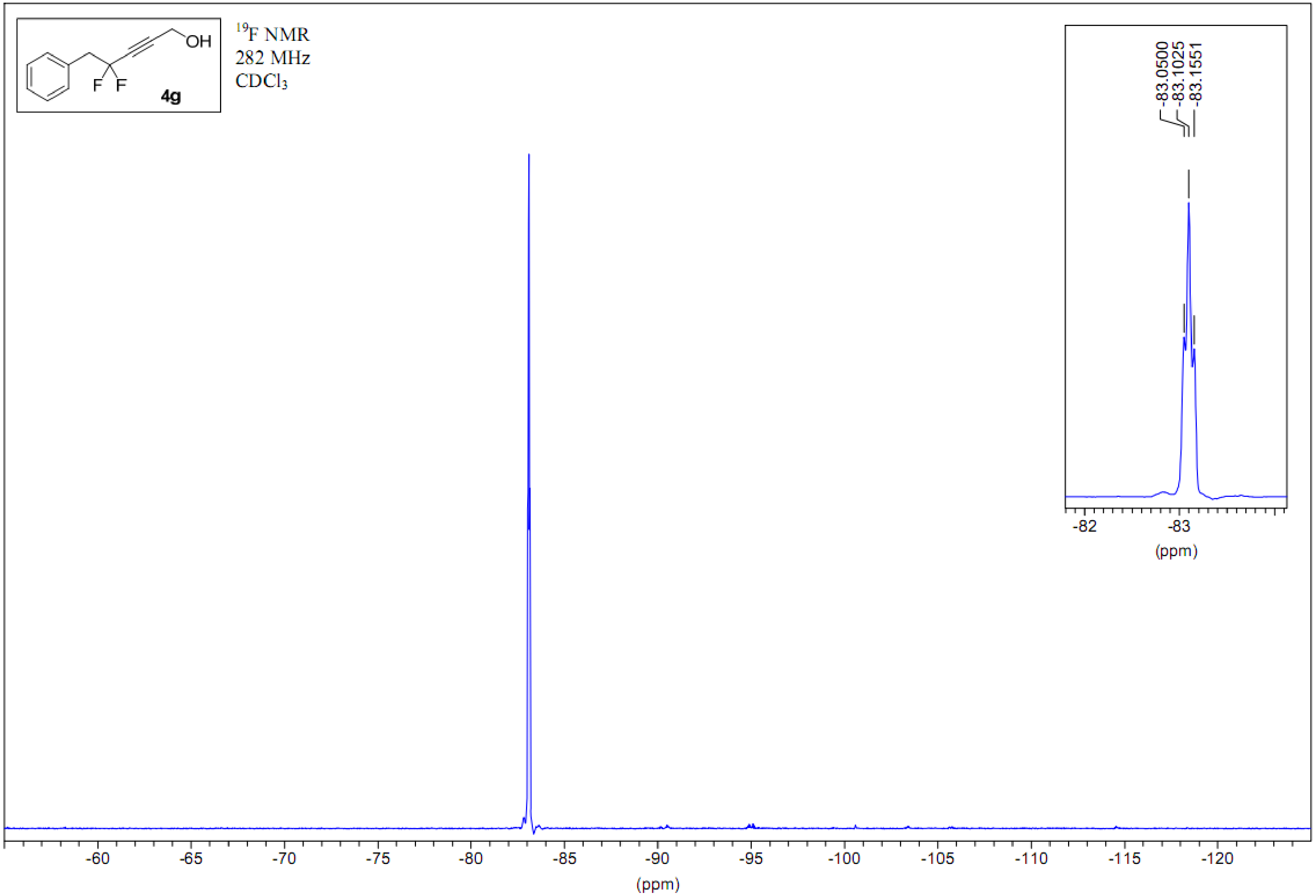
OH
F F
4g
19F NMR
282 MHz
CDCl3
-83.0500
-83.1025
-83.1551
-82
-83
(ppm)
-60
-65
-70
-75
-80
-85
-90
-95
-100
-105
-110
-115
-120
(ppm)

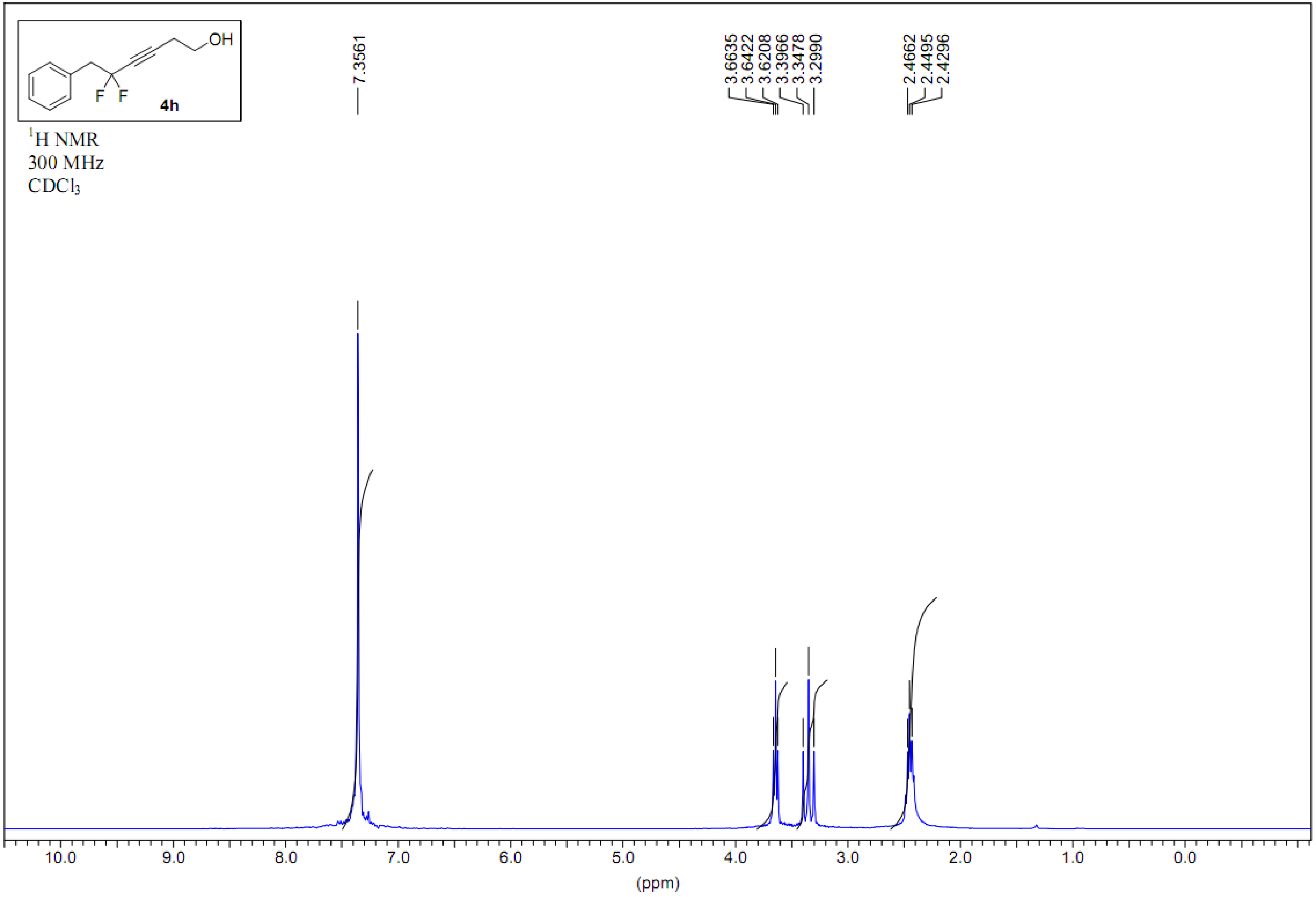
OH
F F
4h
^{1}H NMR
300 MHz
$CDCl_3$
7.3561
3.6635
3.6422
3.6208
3.3966
3.3478
3.2990
2.4662
2.4495
2.4296
10.0
9.0
8.0
7.0
6.0
5.0
4.0
3.0
2.0
1.0
0.0
(ppm)

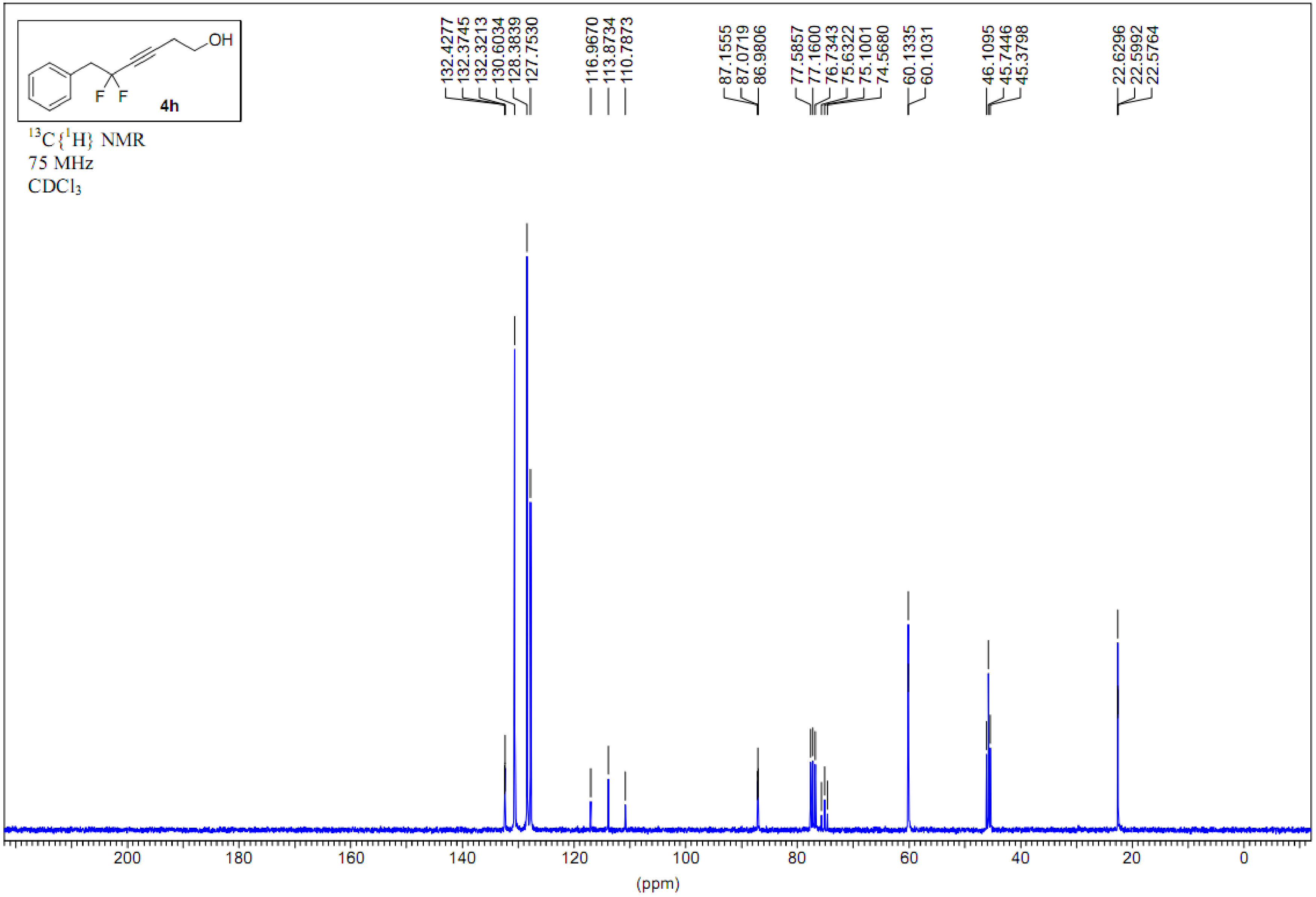

OH
F F
4h
13C{1H} NMR
75 MHz
CDCl3
132.4277
132.3745
132.3213
130.6034
128.3839
127.7530
116.9670
113.8734
110.7873
87.1555
87.0719
86.9806
77.5857
77.1600
76.7343
75.6322
75.1001
74.5680
60.1335
60.1031
46.1095
45.7446
45.3798
22.6296
22.5992
22.5764
200
180
160
140
120
100
80
60
40
20
0
(ppm)

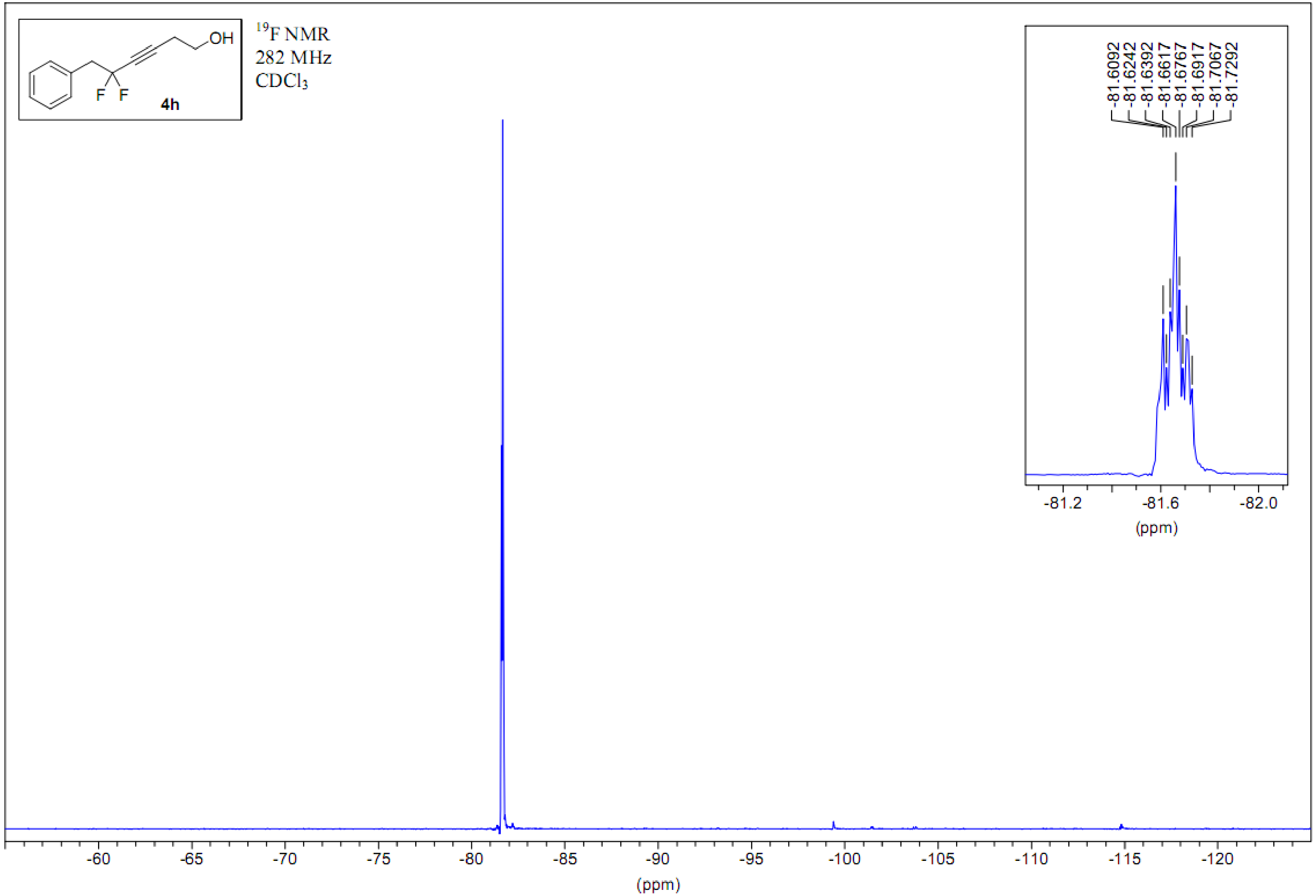

OH
F F
4h
19F NMR
282 MHz
CDCl3
-81.6092
-81.6242
-81.6392
-81.6617
-81.6767
-81.6917
-81.7067
-81.7292
-81.2
-81.6
-82.0
(ppm)
-60
-65
-70
-75
-80
-85
-90
-95
-100
-105
-110
-115
-120
(ppm)

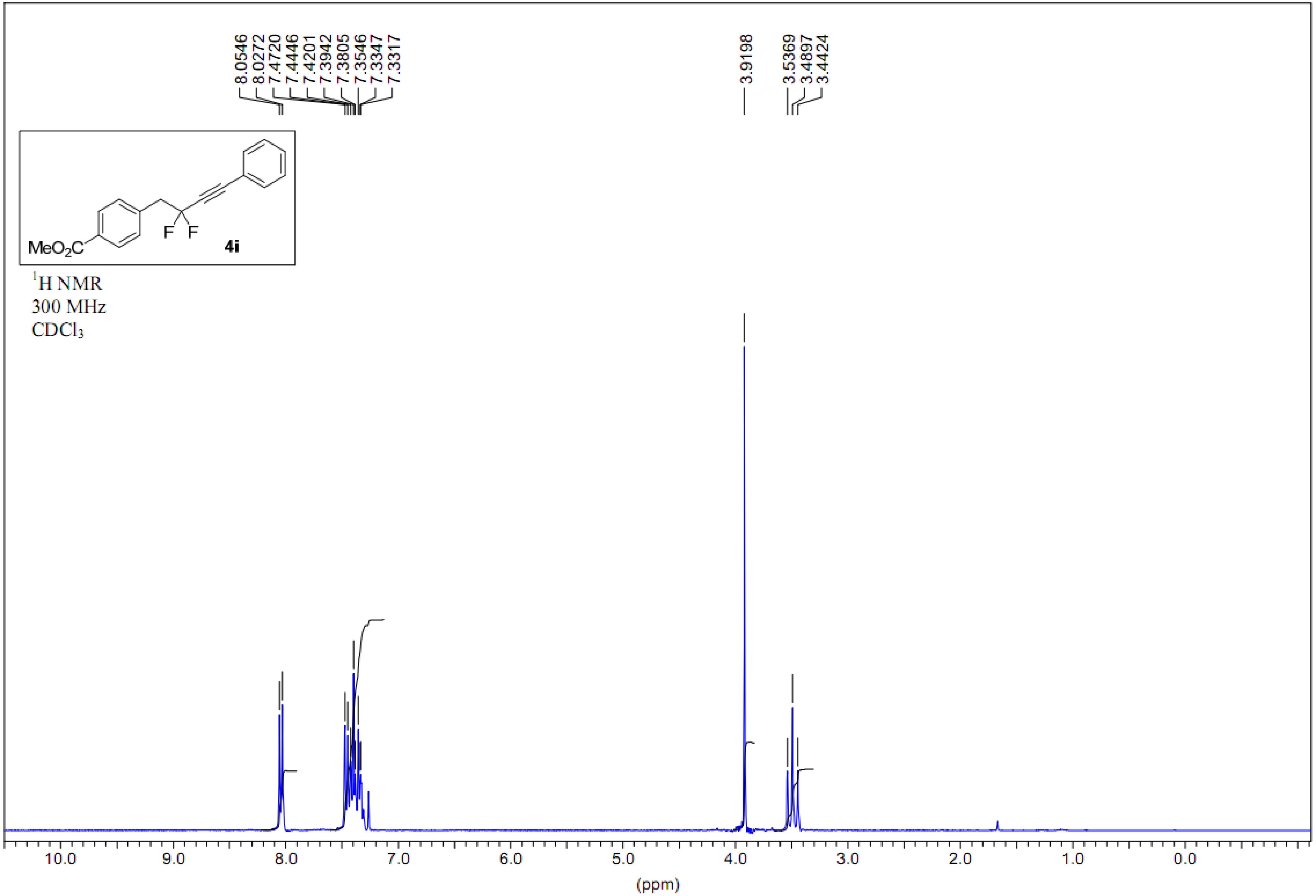
8.0546
8.0272
7.4720
7.4446
7.4201
7.3942
7.3805
7.3546
7.3347
7.3317
3.9198
3.5369
3.4897
3.4424
MeO2C
F F
4i
1H NMR
300 MHz
CDCl3
10.0
9.0
8.0
7.0
6.0
5.0
4.0
3.0
2.0
1.0
0.0
(ppm)

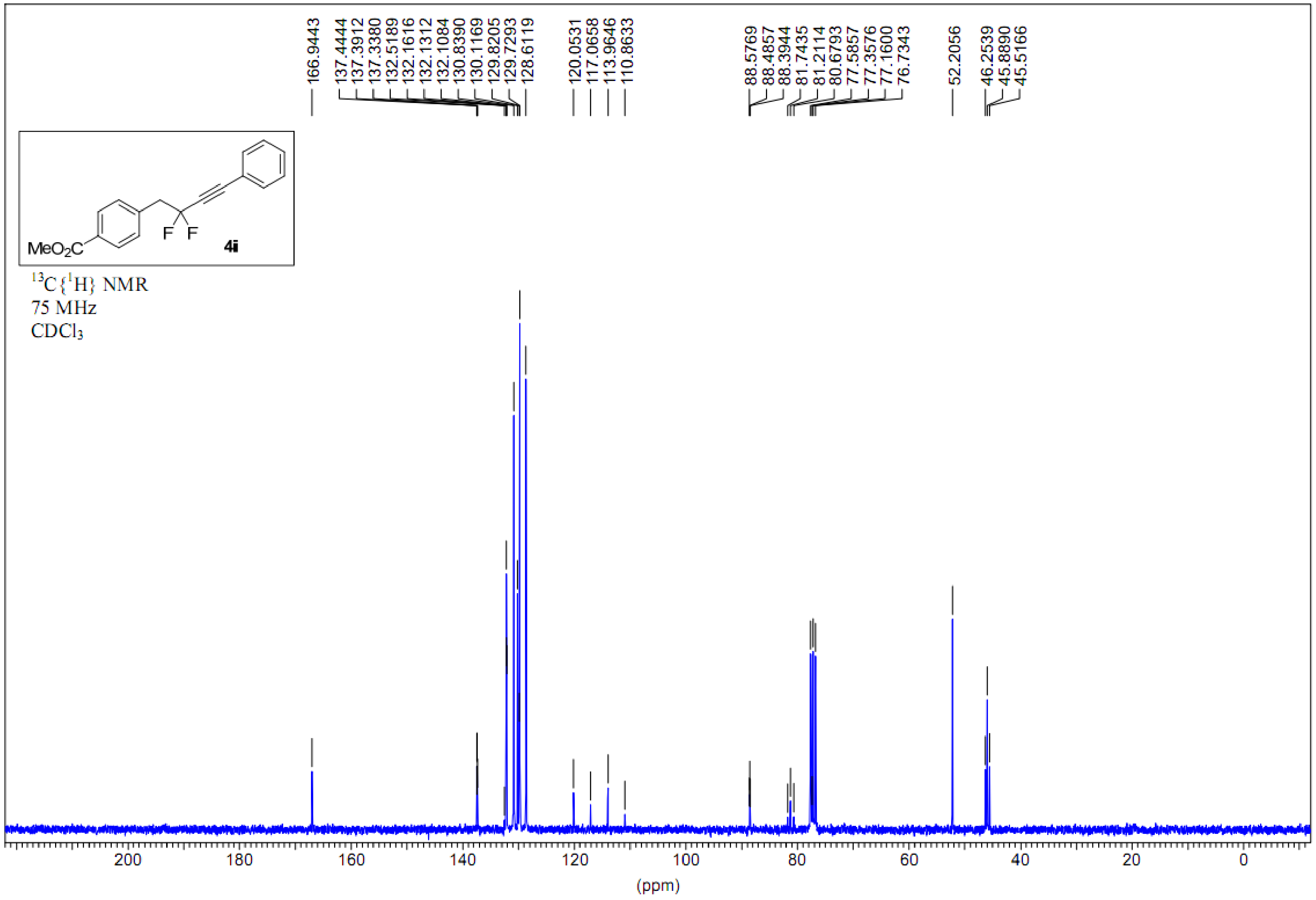
166.9443
137.4444
137.3912
137.3380
132.5189
132.1616
132.1312
132.1084
130.8390
130.1169
129.8205
129.7293
128.6119
120.0531
117.0658
113.9646
110.8633
88.5769
88.4857
88.3944
81.7435
81.2114
80.6793
77.5857
77.3576
77.1600
76.7343
52.2056
46.2539
45.8890
45.5166
MeO2C
F
F
4i
13C{1H} NMR
75 MHz
CDCl3
200
180
160
140
120
100
80
60
40
20
0
(ppm)

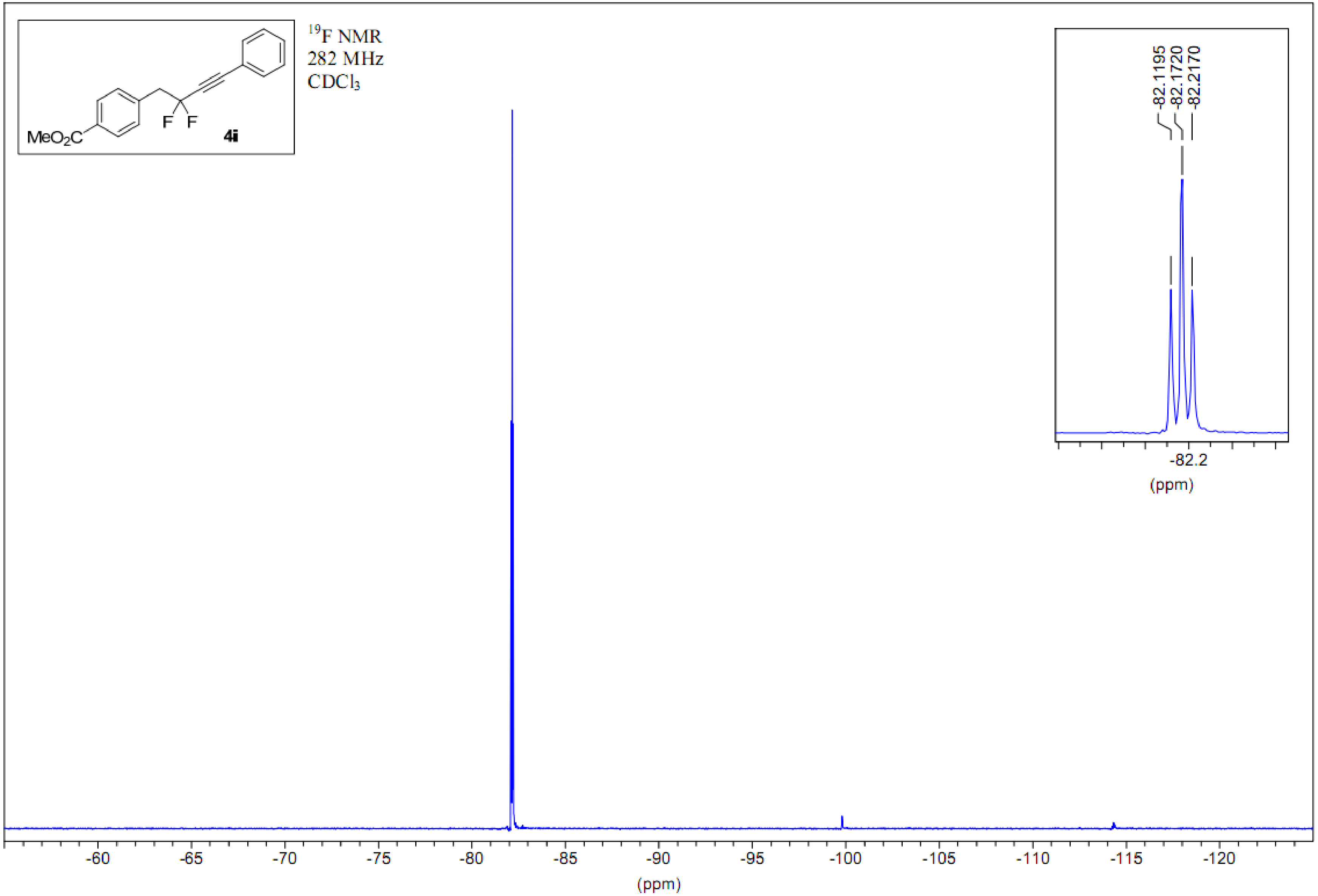
MeO2C
F F
4i
19F NMR
282 MHz
CDCl3
-82.1195
-82.1720
-82.2170
-82.2
(ppm)
-60
-65
-70
-75
-80
-85
-90
-95
-100
-105
-110
-115
-120
(ppm)

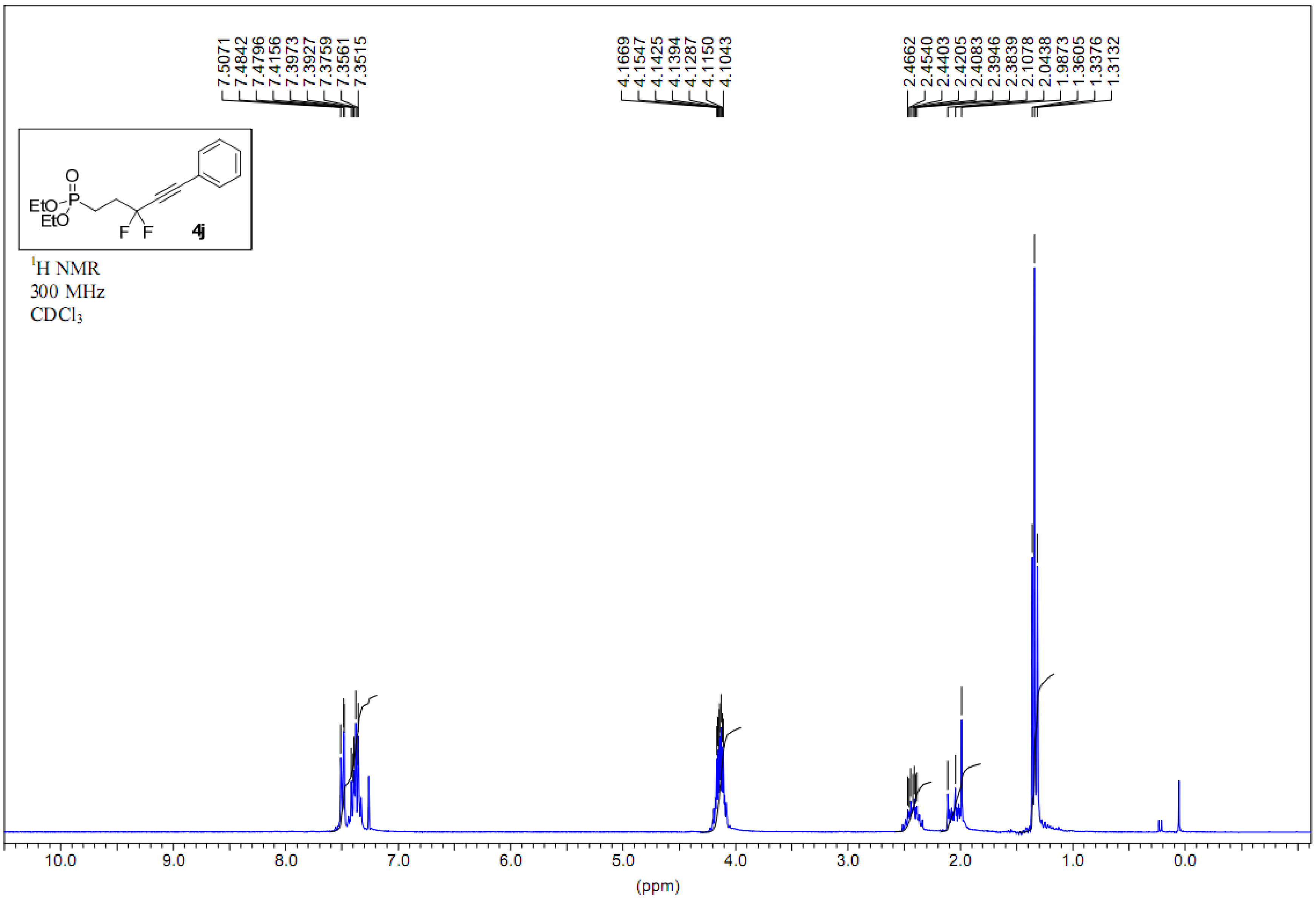

7.5071
7.4842
7.4796
7.4156
7.3973
7.3927
7.3759
7.3561
7.3515
4.1669
4.1547
4.1425
4.1394
4.1287
4.1150
4.1043
2.4662
2.4540
2.4403
2.4205
2.4083
2.3946
2.3839
2.1078
2.0438
1.9873
1.3605
1.3376
1.3132
EtO
EtO
P
O
F
F
4j
^{1}H NMR
300 MHz
CDCl3
10.0
9.0
8.0
7.0
6.0
5.0
4.0
3.0
2.0
1.0
0.0
(ppm)

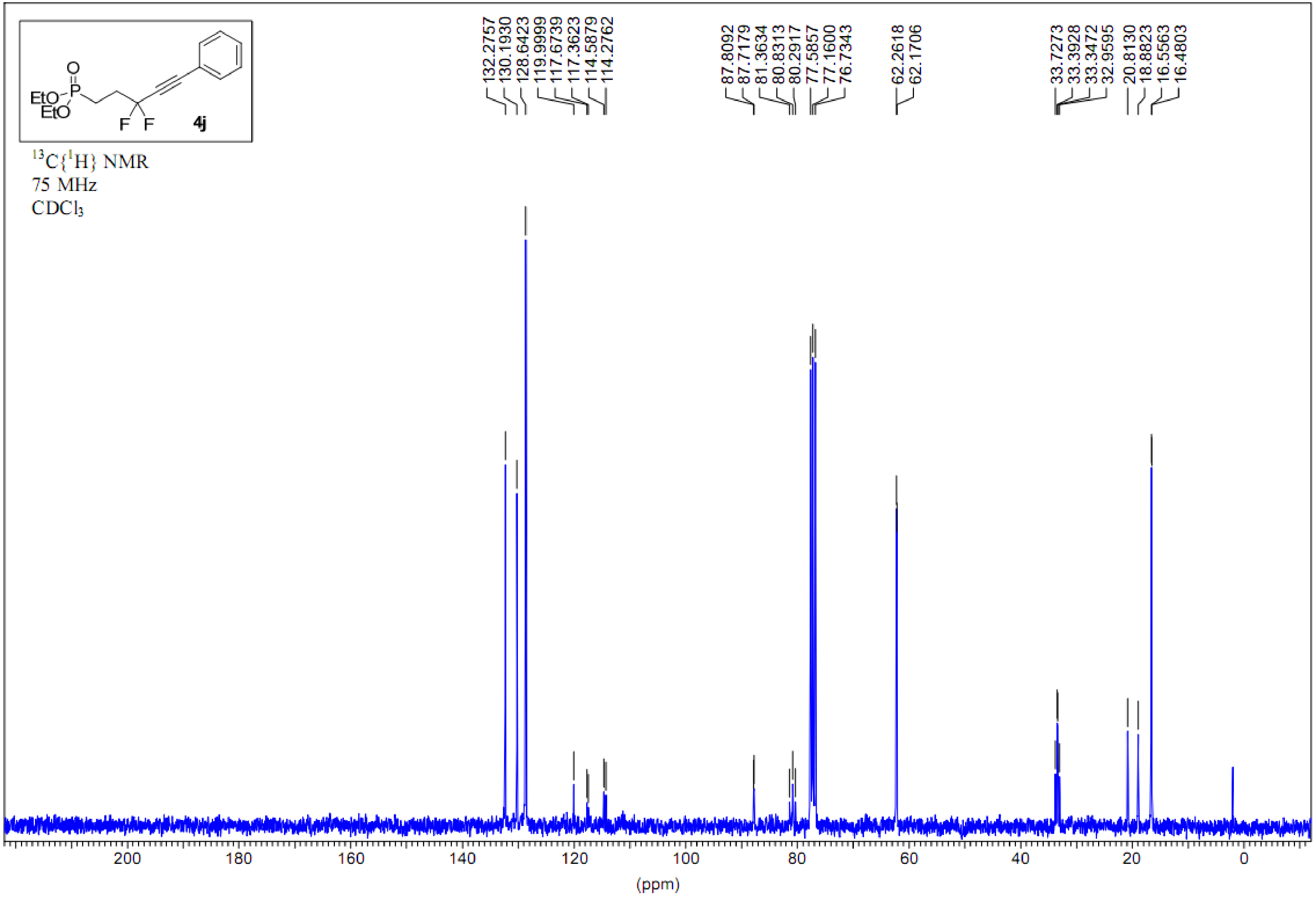
4j
EtO
EtO
P
O
F F
13C{1H} NMR
75 MHz
CDCl3
132.2757
130.1930
128.6423
119.9999
117.6739
117.3623
114.5879
114.2762
87.8092
87.7179
81.3634
80.8313
80.2917
77.5857
77.1600
76.7343
62.2618
62.1706
33.7273
33.3928
33.3472
32.9595
20.8130
18.8823
16.5563
16.4803
200
180
160
140
120
100
80
60
40
20
0
(ppm)

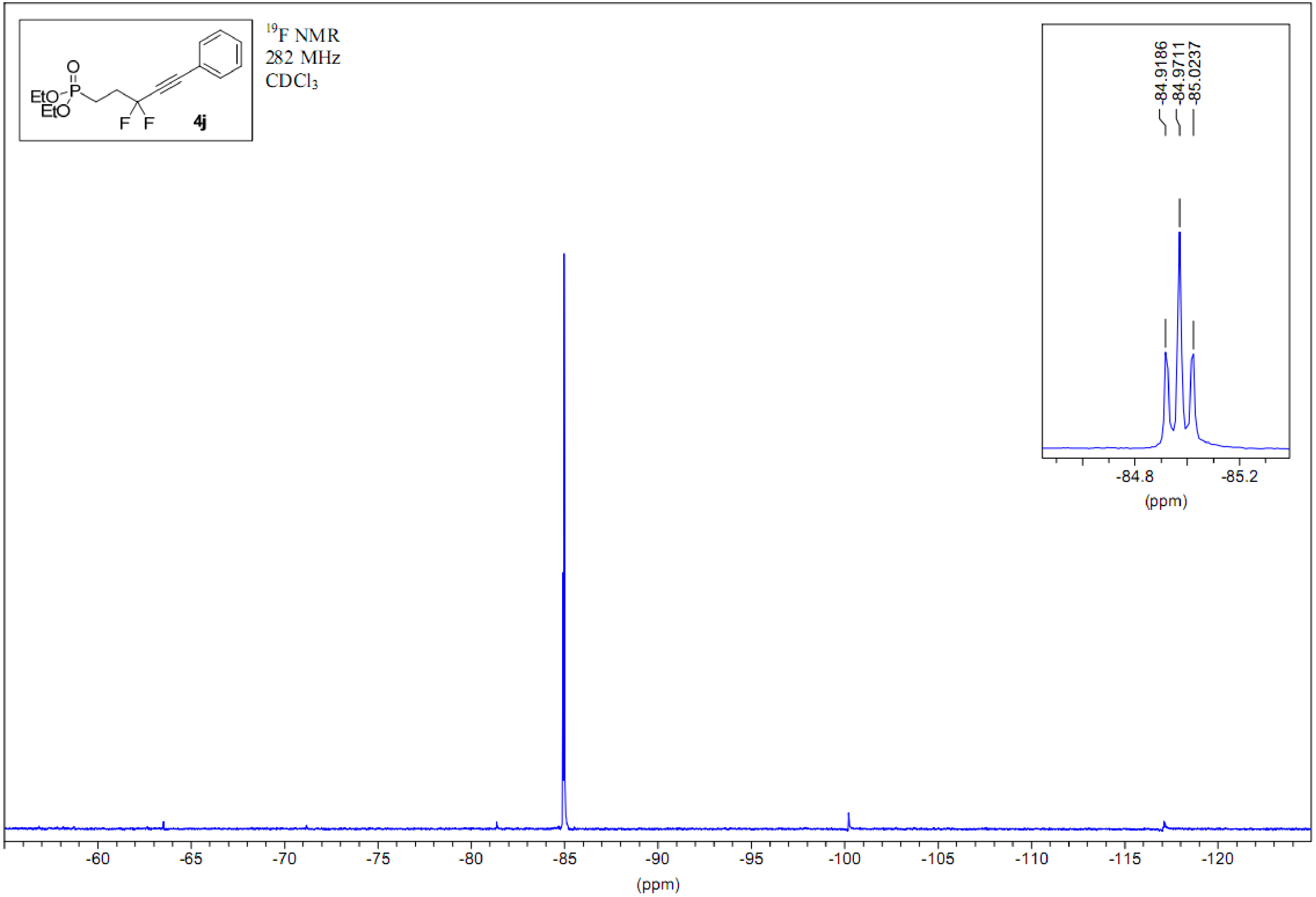

EtO
EtO
O
P
F F
4j
^{19}F NMR
282 MHz
$CDCl_3$
-84.9186
-84.9711
-85.0237
-84.8
-85.2
(ppm)
-60
-65
-70
-75
-80
-85
-90
-95
-100
-105
-110
-115
-120
(ppm)

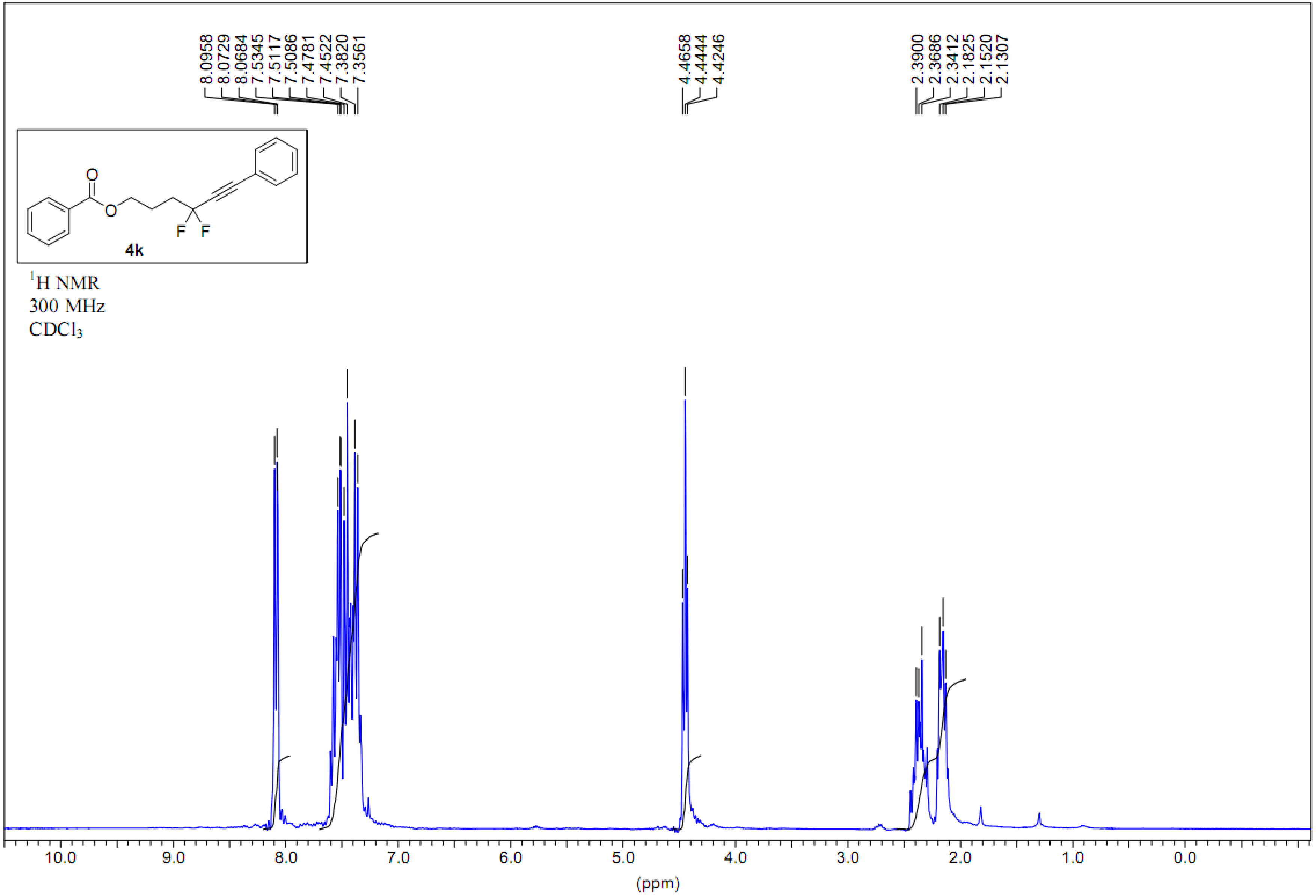
8.0958
8.0729
8.0684
7.5345
7.5117
7.5086
7.4781
7.4522
7.3820
7.3561
4.4658
4.4444
4.4246
2.3900
2.3686
2.3412
2.1825
2.1520
2.1307
O
O
F F
4k
¹H NMR
300 MHz
CDCl3
10.0
9.0
8.0
7.0
6.0
5.0
4.0
3.0
2.0
1.0
0.0
(ppm)

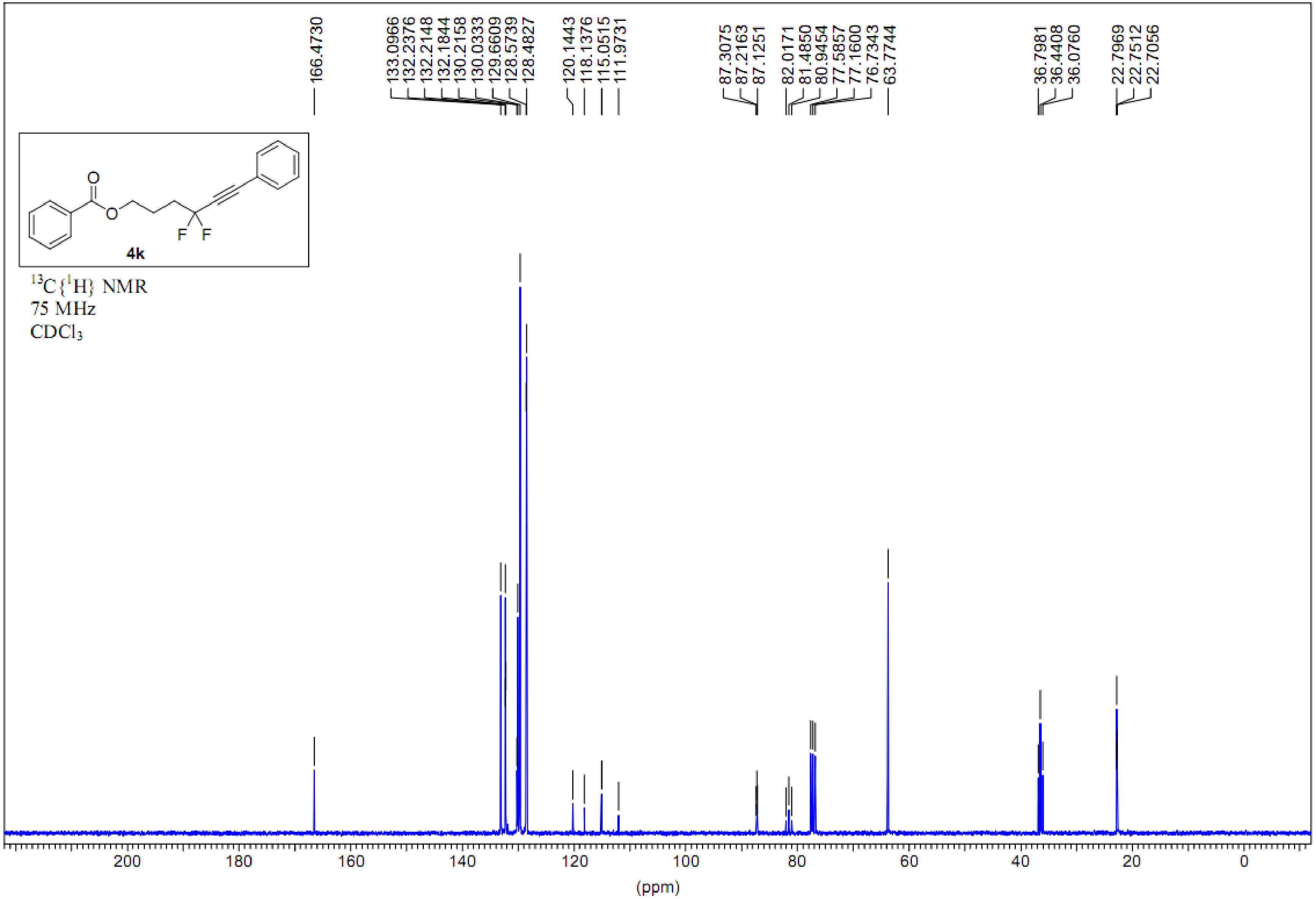
166.4730
133.0966
132.2376
132.2148
132.1844
130.2158
130.0333
129.6609
128.5739
128.4827
120.1443
118.1376
115.0515
111.9731
87.3075
87.2163
87.1251
82.0171
81.4850
80.9454
77.5857
77.1600
76.7343
63.7744
36.7981
36.4408
36.0760
22.7969
22.7512
22.7056
O
O
F
F
4k
$^{13}C\{^{1}H\}$ NMR
75 MHz
$CDCl_3$
200
180
160
140
120
100
80
60
40
20
0
(ppm)

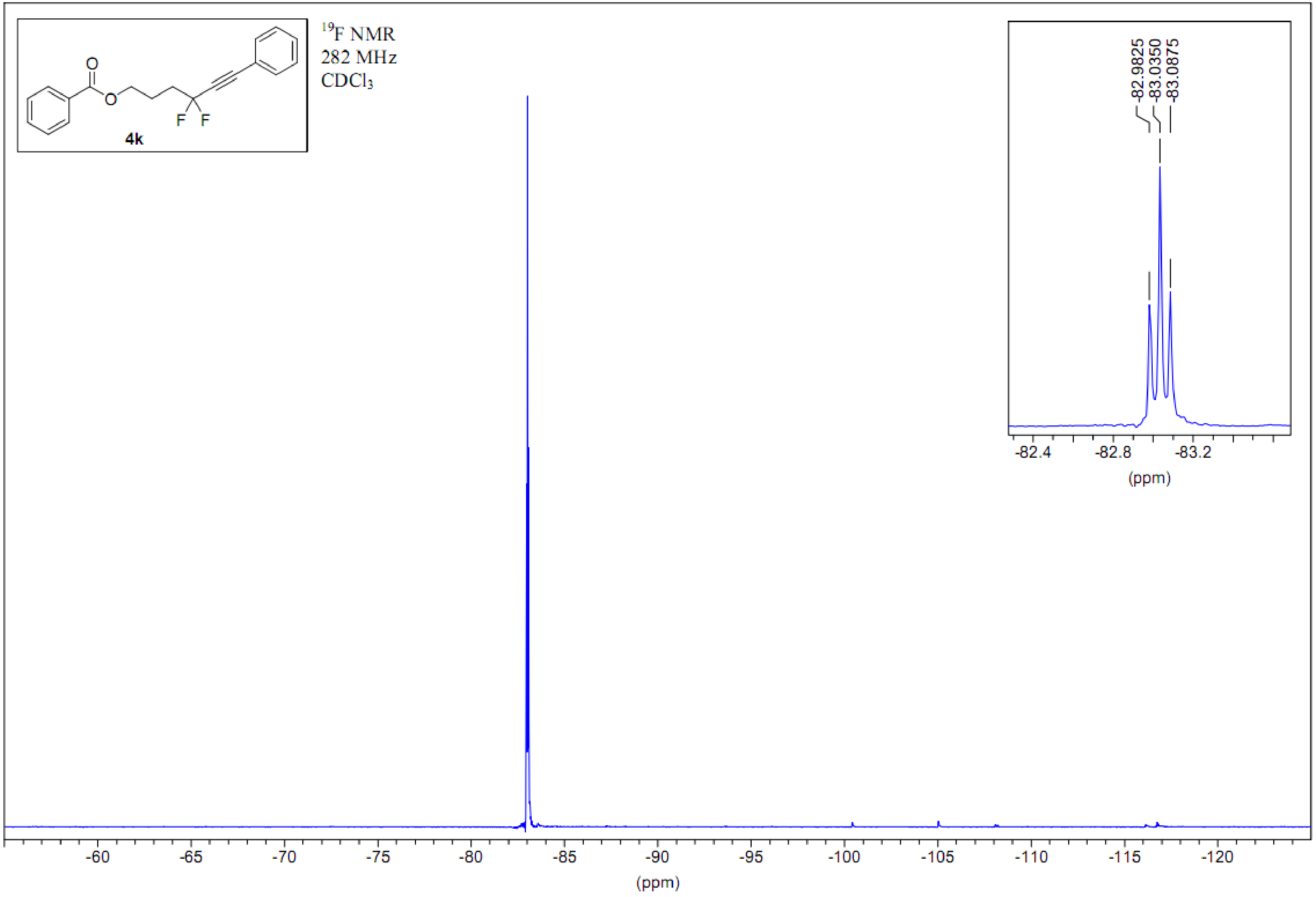

4k
^{19}F NMR
282 MHz
$CDCl_3$
-82.9825
-83.0350
-83.0875
-82.4
-82.8
-83.2
(ppm)
-60
-65
-70
-75
-80
-85
-90
-95
-100
-105
-110
-115
-120
(ppm)

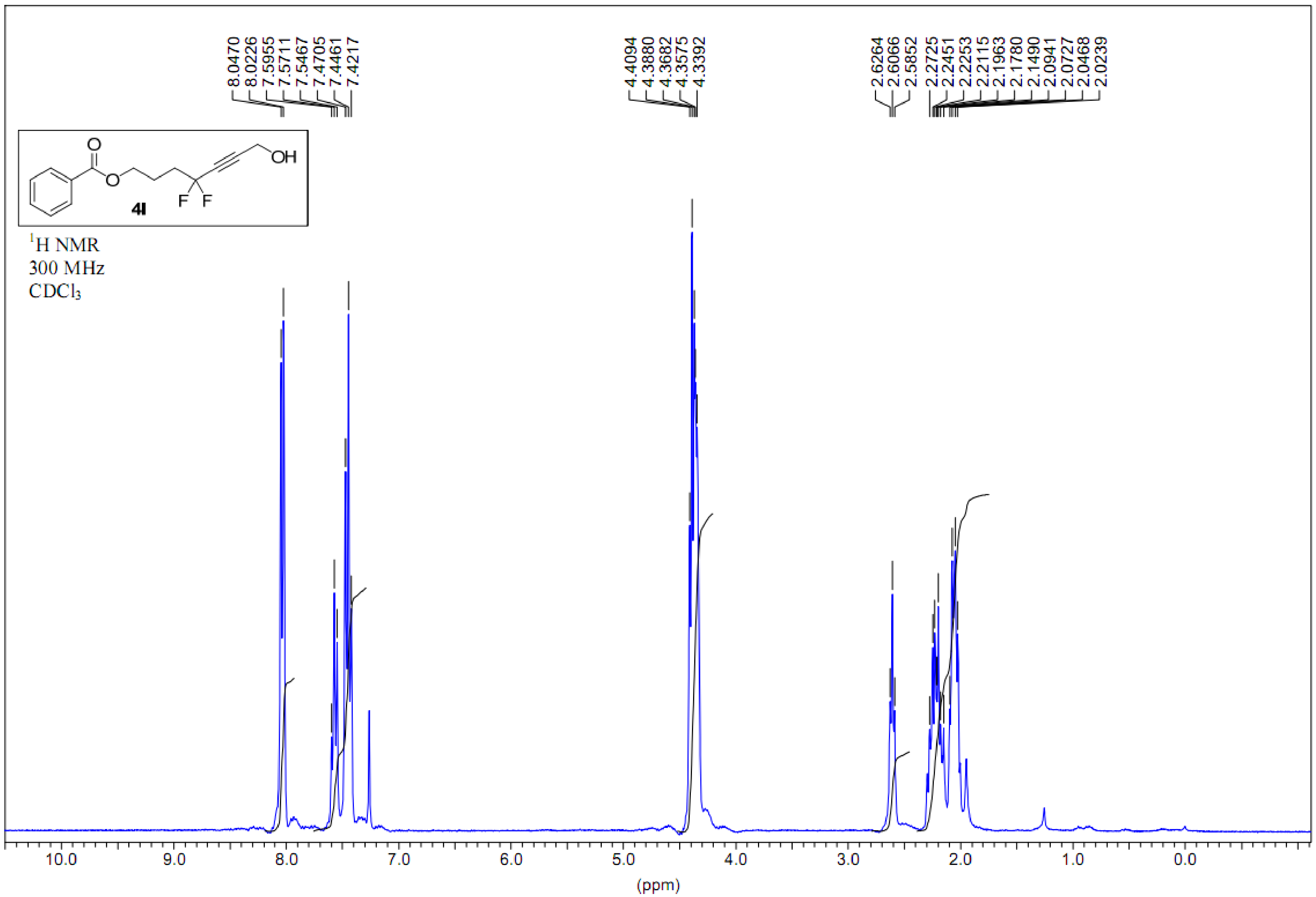

8.0470
8.0226
7.5955
7.5711
7.5467
7.4705
7.4461
7.4217
4.4094
4.3880
4.3682
4.3575
4.3392
2.6264
2.6066
2.5852
2.2725
2.2451
2.2253
2.2115
2.1963
2.1780
2.1490
2.0941
2.0727
2.0468
2.0239
O
O
OH
F F
4l
^{1}H NMR
300 MHz
$CDCl_3$
10.0
9.0
8.0
7.0
6.0
5.0
4.0
3.0
2.0
1.0
0.0
(ppm)

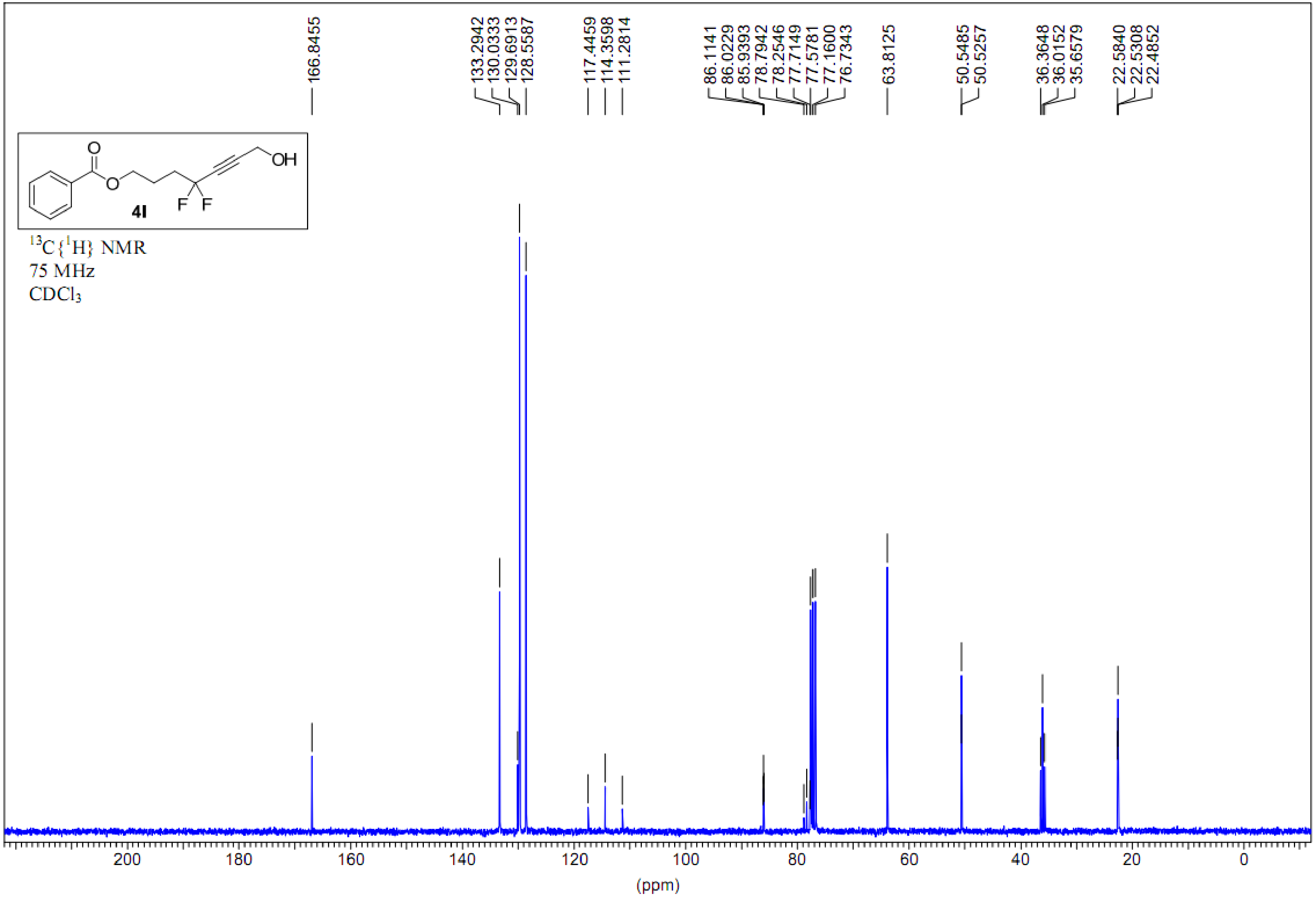

166.8455
133.2942
130.0333
129.6913
128.5587
117.4459
114.3598
111.2814
86.1141
86.0229
85.9393
78.7942
78.2546
77.7149
77.5781
77.1600
76.7343
63.8125
50.5485
50.5257
36.3648
36.0152
35.6579
22.5840
22.5308
22.4852
O
O
OH
F F
4l
$^{13}C\{^{1}H\}$ NMR
75 MHz
$CDCl_3$
200
180
160
140
120
100
80
60
40
20
0
(ppm)

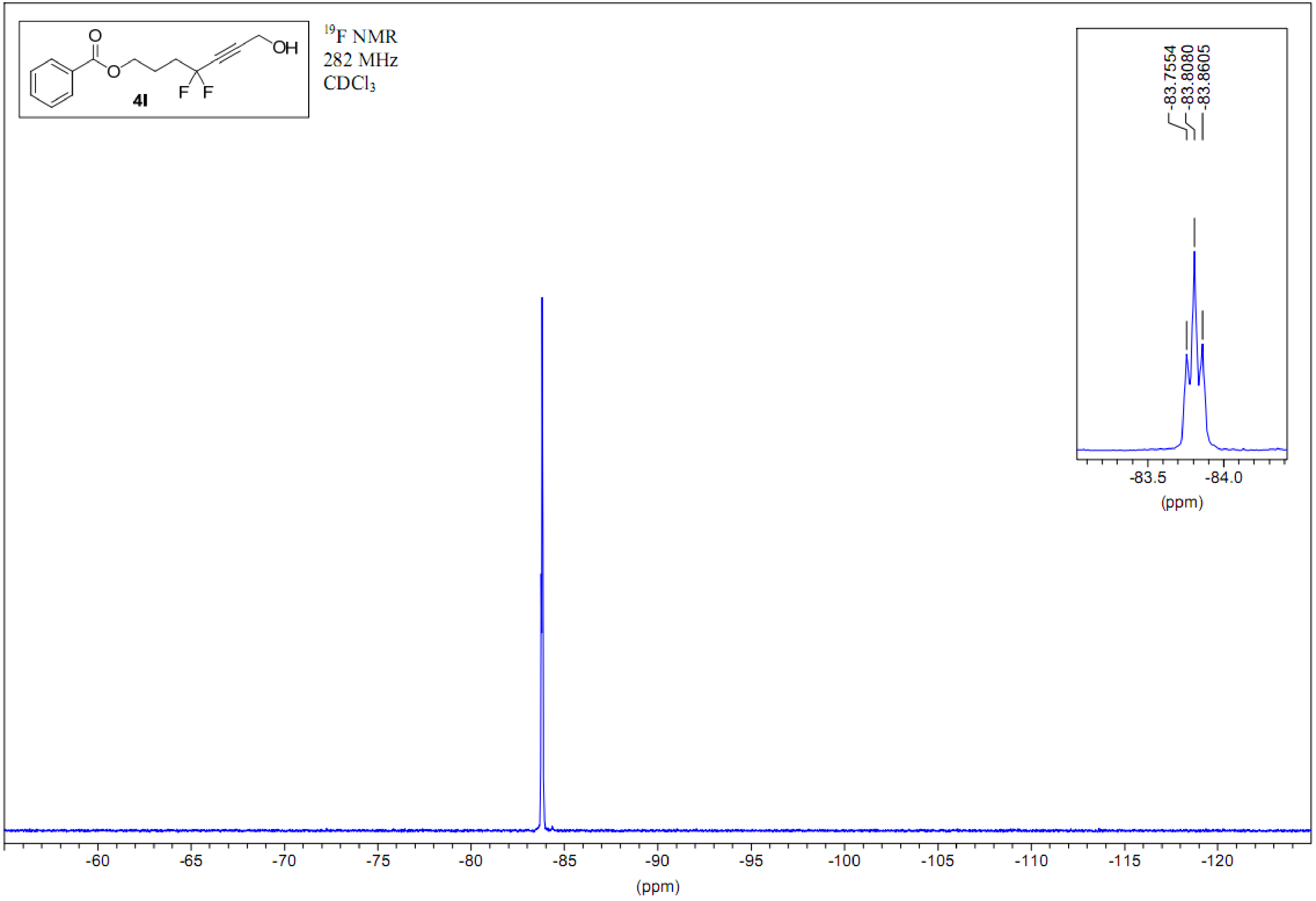

O
O
OH
F F
4l
19F NMR
282 MHz
CDCl3
-83.7554
-83.8080
-83.8605
-83.5
-84.0
(ppm)
-60
-65
-70
-75
-80
-85
-90
-95
-100
-105
-110
-115
-120
(ppm)

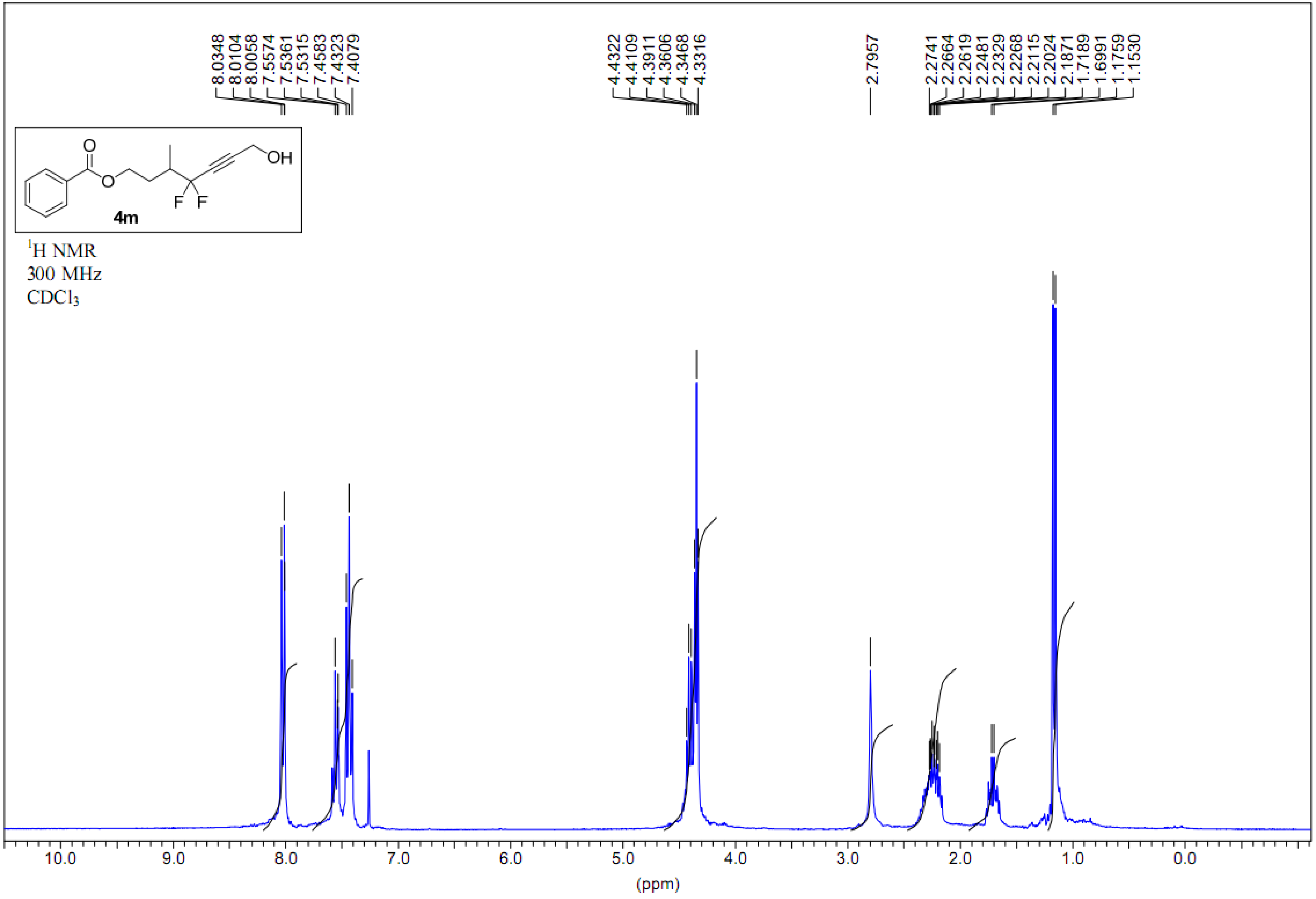
8.0348
8.0104
8.0058
7.5574
7.5361
7.5315
7.4583
7.4323
7.4079
4.4322
4.4109
4.3911
4.3606
4.3468
4.3316
2.7957
2.2741
2.2664
2.2619
2.2481
2.2329
2.2268
2.2115
2.2024
2.1871
1.7189
1.6991
1.1759
1.1530
O
O
OH
F F
4m
1H NMR
300 MHz
CDCl3
10.0
9.0
8.0
7.0
6.0
5.0
4.0
3.0
2.0
1.0
0.0
(ppm)

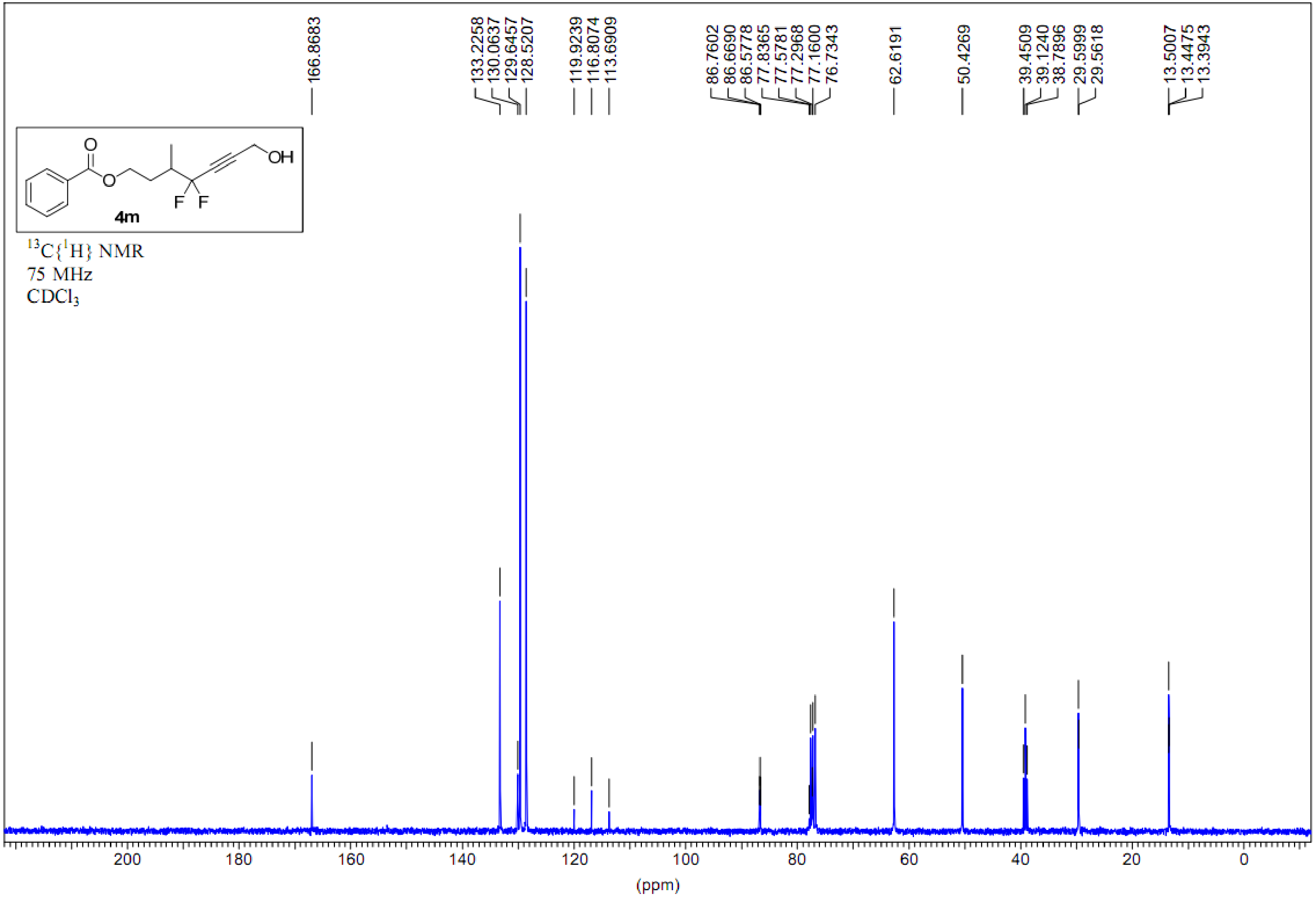
4m
O
O
OH
F F
$^{13}C\{^1H\}$ NMR
75 MHz
$CDCl_3$
166.8683
133.2258
130.0637
129.6457
128.5207
119.9239
116.8074
113.6909
86.7602
86.6690
86.5778
77.8365
77.5781
77.2968
77.1600
76.7343
62.6191
50.4269
39.4509
39.1240
38.7896
29.5999
29.5618
13.5007
13.4475
13.3943
200
180
160
140
120
100
80
60
40
20
0
(ppm)

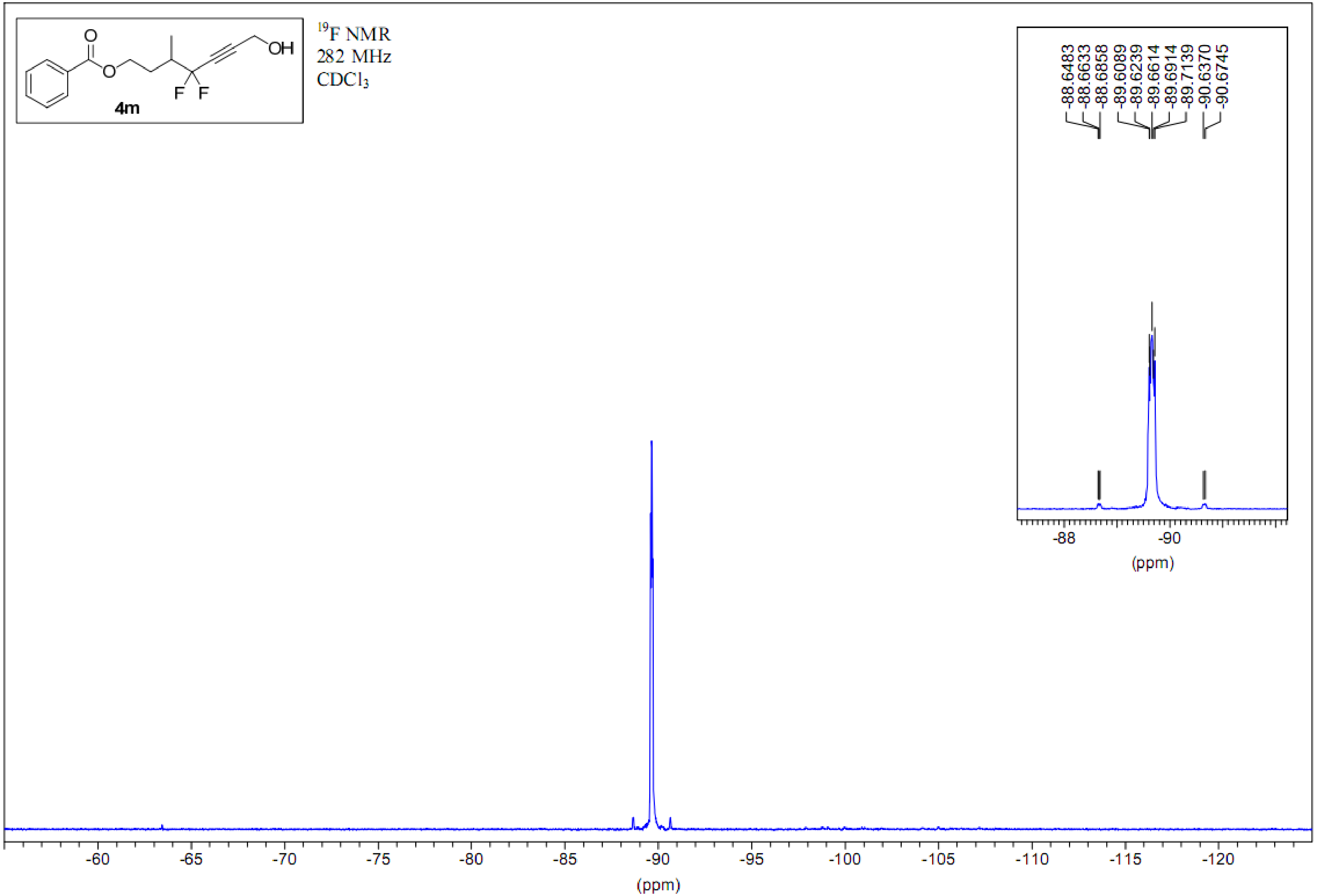
4m
F F
OH
O
19F NMR
282 MHz
CDCl3
-88.6483
-88.6633
-88.6858
-89.6089
-89.6239
-89.6614
-89.6914
-89.7139
-90.6370
-90.6745
-88
-90
(ppm)
-60
-65
-70
-75
-80
-85
-90
-95
-100
-105
-110
-115
-120
(ppm)
